# The CHK1 inhibitor MU380 significantly increases the sensitivity of human docetaxel‐resistant prostate cancer cells to gemcitabine through the induction of mitotic catastrophe

**DOI:** 10.1002/1878-0261.12756

**Published:** 2020-07-16

**Authors:** Stanislav Drápela, Prashant Khirsariya, Wytske M. van Weerden, Radek Fedr, Tereza Suchánková, Diana Búzová, Jan Červený, Aleš Hampl, Martin Puhr, William R. Watson, Zoran Culig, Lumír Krejčí, Kamil Paruch, Karel Souček

**Affiliations:** ^1^ Department of Cytokinetics Institute of Biophysics of the Czech Academy of Sciences Brno Czech Republic; ^2^ International Clinical Research Center Center for Biomolecular and Cellular Engineering St. Anne's University Hospital in Brno Czech Republic; ^3^ Department of Experimental Biology Faculty of Science Masaryk University Brno Czech Republic; ^4^ Department of Chemistry, CZ Openscreen Faculty of Science Masaryk University Brno Czech Republic; ^5^ Department of Urology Erasmus MC Cancer Institute Erasmus University Medical Center Rotterdam The Netherlands; ^6^ Department of Adaptive Biotechnologies Global Change Research Institute of the Czech Academy of Sciences Brno Czech Republic; ^7^ Department of Histology and Embryology Faculty of Medicine Masaryk University Brno Czech Republic; ^8^ Department of Urology Experimental Urology Medical University of Innsbruck Austria; ^9^ School of Medicine Conway Institute of Biomolecular and Biomedical Research University College Dublin Ireland; ^10^ Department of Biology Faculty of Medicine Masaryk University Brno Czech Republic; ^11^ National Centre for Biomolecular Research Masaryk University Brno Czech Republic

**Keywords:** castration‐resistant prostate cancer, checkpoint kinase 1, docetaxel resistance, gemcitabine, mitotic catastrophe, MU380

## Abstract

As treatment options for patients with incurable metastatic castration‐resistant prostate cancer (mCRPC) are considerably limited, novel effective therapeutic options are needed. Checkpoint kinase 1 (CHK1) is a highly conserved protein kinase implicated in the DNA damage response (DDR) pathway that prevents the accumulation of DNA damage and controls regular genome duplication. CHK1 has been associated with prostate cancer (PCa) induction, progression, and lethality; hence, CHK1 inhibitors SCH900776 (also known as MK‐8776) and the more effective SCH900776 analog MU380 may have clinical applications in the therapy of PCa. Synergistic induction of DNA damage with CHK1 inhibition represents a promising therapeutic approach that has been tested in many types of malignancies, but not in chemoresistant mCRPC. Here, we report that such therapeutic approach may be exploited using the synergistic action of the antimetabolite gemcitabine (GEM) and CHK1 inhibitors SCH900776 and MU380 in docetaxel‐resistant (DR) mCRPC. Given the results, both CHK1 inhibitors significantly potentiated the sensitivity to GEM in a panel of chemo‐naïve and matched DR PCa cell lines under 2D conditions. MU380 exhibited a stronger synergistic effect with GEM than clinical candidate SCH900776. MU380 alone or in combination with GEM significantly reduced spheroid size and increased apoptosis in all patient‐derived xenograft 3D cultures, with a higher impact in DR models. Combined treatment induced premature mitosis from G1 phase resulting in the mitotic catastrophe as a prestage of apoptosis. Finally, treatment by MU380 alone, or in combination with GEM, significantly inhibited tumor growth of both PC339‐DOC and PC346C‐DOC xenograft models in mice. Taken together, our data suggest that metabolically robust and selective CHK1 inhibitor MU380 can bypass docetaxel resistance and improve the effectiveness of GEM in DR mCRPC models. This approach might allow for dose reduction of GEM and thereby minimize undesired toxicity and may represent a therapeutic option for patients with incurable DR mCRPC.

## Graphical abstract

As the treatment options for metastatic castration‐resistant prostate cancer (mCRPC) are limited, novel therapeutic approaches are urgently needed. Here, we showed that checkpoint kinase 1 (CHK1) inhibitor MU380 significantly potentiates gemcitabine cytotoxicity in docetaxel‐resistant mCRPC models, resulting in mitotic catastrophe. These data provide a preclinical rationale for the application of MU380 in a clinical setting for the therapy of mCRPC.

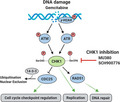

**Abbreviations**
AR: androgen receptor
ATM: ataxia‐telangiectasia mutated
ATR: ataxia‐telangiectasia and Rad3‐related
CHK1: checkpoint kinase 1
CHK2: checkpoint kinase 2
DAPI: 4′,6‐diamidin‐2‐phenylindol
DDR: DNA damage response
GEM: gemcitabine
IC_50_: half‐maximal inhibitory concentration
mCRPC: metastatic castration‐resistant prostate cancer
MFI: median fluorescence index
mpk: milligram per kilogram body weight
PCa: prostate cancer
PDX: patient‐derived xenograft
PE: phycoerythrin
pH2AX: phosphorylated γH2A.X
pHH3: phosphorylated histone H3
PI: propidium iodide
RFU: relative fluorescence unit
RLU: relative luminescence unit
SHO: SCID (severe combined immunodeficient) hairless outbred
TMRE: tetramethylrhodamine, ethyl ester

## 1 Introduction

Prostate cancer (PCa) represents one of the most heterogeneous and clinically common malignancies in men. Despite a high initial effectivity of androgen deprivation therapy in localized disease with medium and high risk, nearly half of the patients experience progression to the incurable and lethal form termed metastatic castration‐resistant PCa (mCRPC) [1]. Treatment options for this advanced stage of the disease are rather limited. Specifically, docetaxel has been used as the most effective treatment strategy for mCRPC patients since 2004. Nevertheless, it gives only modest survival benefit with most patients invariably progressing due to acquired or inherent drug resistance [2, 3]. Due to the very low efficacy of chemotherapeutics, prolonged anamnesis and resistance, the follow‐up therapies may pose more risk than help, indicating that identification of new druggable targets in mCRPC is crucial for the development of more efficient therapies.

DNA damaging therapy triggers various cellular processes including DNA damage response (DDR), cell cycle arrest, DNA repair, or apoptosis [4]. The clinical genomics study of advanced PCa has demonstrated that alterations in DDR genes such as loss of BRCA1/2 or p53 function are present in almost one‐fourth of all mCRPC cases [5, 6, 7]. Checkpoint kinase 1 (CHK1) is a highly conserved protein kinase that is activated at replication fork by single‐stranded DNA or bulky DNA lesions [8] to prevent cell cycle progression and recruit the DNA repair machinery to damaged sites *via* CHK1‐dependent Rad51 phosphorylation [9, 10, 11, 12]. CHK1 acts as a distal transducer in the core DDR signaling network ataxia‐telangiectasia and Rad3‐related (ATR)‐CHK1 which along with ataxia‐telangiectasia mutated (ATM)‐CHK2‐p53 govern genomic stability and prevent malignant transformations [13, 14, 15]. In cancer, the principal activator of the ATR‐CHK1 pathway is replication stress that is a consequence of activated oncogenes and dysfunctional G1/S checkpoint control [16]. Interestingly, androgen receptor (AR) signaling has been reported to specifically regulate DDR genes and its activity strongly correlates with the enhanced activation of ATR‐CHK1 axis, castration resistance, metastasis, and decreased survival of PCa patients [17, 18]. Given the high‐rate mutation events in DDR in mCRPC, CHK1 remains an essential molecule for controlling DDR and cell cycle and its targeting represents a particularly intriguing strategy for anticancer therapy [19, 20].

In our previous study, we reported the discovery of the novel potent and selective CHK1 inhibitor MU380 [19]. This small molecule possesses a highly unusual *N*‐trifluoromethylpyrazole motif that renders the molecule more metabolically robust to oxidative *N*‐dealkylation, which is reflected in the compound's favorable *in vivo* properties.

A combination of MU380 and gemcitabine (GEM) induces higher accumulation of DNA damage following increased cell death in a variety of cancer cell lines and is more effective in an *in vivo* mouse xenograft model [19] than GEM plus the clinical candidate SCH900776 [21]. Our recent study also demonstrated that MU380 can sensitize lymphoid cancer cells to cytotoxic chemotherapeutic drugs such as GEM and fludarabine and that MU380 is effective as a single agent in models with defective *TP53* function [21]. Here, we report a comprehensive investigation of the single‐agent efficacy of MU380 and its ability to potentiate the effect of GEM in various resistant PCa models. MU380 effectively sensitized all naïve and docetaxel‐resistant (DR) cell lines by selective inhibition of GEM‐induced CHK1 autophosphorylation of Ser296. While MU380 monotherapy showed significant efficacy in DR mCRPC PCa patient‐derived xenografts (PDX), importantly, combined treatment with GEM resulted in significant tumor regression in the PC339‐DOC and PC346C‐DOC xenografts, with observed efficacy of MU380 monotherapy in PC339‐DOC. Altogether, the data provide an attractive preclinical rationale for further clinical investigation of CHK1 inhibitors in the context of eradication of aggressive, incurable mCRPC.

## 2 Material and methods

### 2.1 Cell lines, xenografts, and chemicals

Docetaxel‐resistant DU145 and PC3 PCa cell lines (indicated by no. 1) were derived as previously reported [22]. Docetaxel resistance was maintained by a continuous supply of docetaxel (Cell Signaling, Danvers, MA, USA) in the final concentration of 12.5 nm. DR DU145 and PC3 from Dublin (indicated by no. 2) were generated as described previously [23]. Docetaxel resistance was retained by the addition of 12 nm docetaxel monthly. All cell lines were maintained at 37 °C (5% CO_2_) in RPMI 1640 (Thermofisher Scientific, Waltham, MA, USA) media supplemented with 10% FBS and 100 U·mL^−1^ penicillin/streptomycin. The chemotherapy‐naïve PC346C and PC339 xenografts and their DR derivatives PC346C‐DOC or PC339‐DOC, respectively, were established as described previously [24, 25]. GEM was purchased from Carbosynth Ltd (Compton, UK). All cell models were routinely tested for mycoplasma contamination. Cells were authenticated using AmpFLSTR Identifiler Plus PCR Amplification Kit (Thermofisher Scientific) to verify their origin. CHK1 inhibitors SCH900776 (currently in the second phase of clinical trials) and novel MU380 (preclinical studies) were synthesized as previously published [19].

### 2.2 Drug treatments

Cells were seeded and allowed to attach overnight. Attached cells were treated with different concentrations of GEM for 24 h, followed by the addition of CHK1 inhibitors (either SCH900776 or MU380) for 2 h. Thereafter, the cells were replenished with fresh medium and harvested for appropriate assay at the indicated time points.

### 2.3 Cell proliferation assay

Cell proliferation was assessed by CyQUANT™ Cell Proliferation Assay (Thermofisher Scientific). Wide‐spectra drug screening was performed on 384‐well plates (Corning, NY, USA). The cells were seeded in the density of 20 000 cells·cm^−2^ and cultivated for 24 h. Next, the treatment by the range of concentrations of all drugs was performed with EpMotion® 5075 Automated Liquid Handling System (Eppendorf, Hamburg, Germany), and cells were cultivated for the next 48 h. CyQUANT™ Cell Proliferation Assay was performed in the endpoint to analyze cell proliferation. For the combined treatment analysis, the cells were seeded in the density of 20 000 cells·cm^−2^ into 96‐well plates (Corning). Twenty‐four hours later, the cells were treated with GEM (in MQ water) concentration range for 24 h. The next day, the CHK1 inhibitors SCH900776 (4 µm in DMSO) or MU380 (4 µm in DMSO) were added for 2 h, followed by complete media exchange. CyQuant assay was performed 48 h post‐treatments as recommended by the manufacturer. The fluorescence was detected at 520 nm on a plate reader Fluostar Galaxy (BMG Labtech, Ortenberg, Germany).

### 2.4 3D spheroid assay

Ten thousand cells per well were seeded into ultra‐low attachment 384‐well plates (Corning) in the volume of 50 µL of media, the plates were centrifuged (10 min, 200 ***g***) and cells allowed to proliferate and form spheroids in 48 h. After that time, the spheroids were pretreated with GEM (0.25 or 0.5 µm in fresh media) for 24 h followed by SCH900776 (4 µm in fresh media) or MU380 (4 µm in fresh media) treatment. Forty‐eight hours later, cell viability was determined by CellTiter‐Glo luminescent cell viability assay (Promega, Madison, WI, USA) according to the manufacturer's recommendations. Luminescence was monitored at 560 nm using plate reader Infinite 200 PRO (Tecan, Männedorf, Switzerland). Calcein AM and propidium iodide (PI) were applied for the fluorescent analysis of viable and dead cells using ImageXpress Micro XLS Widefield High‐Content Analysis System (Molecular Devices, San Jose, CA, USA). The spheroid size was determined by the quantification of transmitted light, and fluorescent signal was quantified using high‐content image analysis software metaxpress (Molecular Devices).

### 2.5 Immunostaining

For immunofluorescence, cells were washed, fixed in 4% paraformaldehyde, permeabilized in 0.25% Triton X‐100, and blocked in 3% BSA containing 0.1% Triton X‐100. Afterward, the cells were stained in suspension with biotin‐conjugated primary antiphospho‐histone H3 followed by streptavidin‐phycoerythrin (PE) secondary antibody, anti‐α‐tubulin followed by mouse Alexa Fluor 647‐conjugated secondary antibody and 4′,6‐diamidin‐2‐phenylindol (DAPI) for nuclear localization. Cells were then washed twice, mounted in Mowiol 4‐88 (MilliporeSigma, Burlington, MA, USA) + 0.6% 1,4‐diazabicyclo[2.2.2]octane (Sigma‐Aldrich, St. Louis, MO, USA) as an antifade agent and let dry for 2 h at 37 °C. Slides were analyzed by Olympus FV10i scanning microscope using a 60× objective. Material, clones, dilutions, catalog numbers, and producers are listed in Table S3.

### 2.6 Immunoblotting

Cells were washed in PBS and harvested in radioimmunoprecipitation assay buffer (Table S3) enriched with protease inhibitors (Serva, Heidelberg, Germany) and phosphatase inhibitor cocktail (MilliporeSigma). The protein concentration was determined using a DC (detergent‐compatible) protein assay (Bio‐Rad, Hercules, CA, USA). The cell lysates were diluted to the same concentrations and mixed with loading buffer (150 mmol·L^−1^ Tris–HCl pH 6.8, 3% SDS, 0.03% bromophenol blue, 30% glycerol, 3% β‐mercaptoethanol). Equivalent protein quantities were separated by SDS/PAGE and transferred onto polyvinylidene difluoride membranes (MilliporeSigma). The membranes were blocked in Tris‐buffered saline (TBS) containing 0.1% Tween‐20 and 5% nonfat dry milk for 1 h. The membranes were washed with TBS–Tween and incubated with specific primary antibodies overnight at 4 °C. The following primary antibodies were used: CHK1, pCHK1 (S296), pCHK1 (S345), phosphorylated γH2A.X (pH2AX; S139), and β‐actin. The membranes were washed and then incubated with secondary anti‐mouse IgG or anti‐rabbit IgG (GE Healthcare, Chicago, IL, USA) antibodies for 1 h. Detection of antibody reactivity was performed using chemiluminescence substrate Immobilon Western HRP Substrate (MilliporeSigma) and ChemiDoc™ Imaging System (Bio‐Rad). Dilutions, catalog numbers, and producers are listed in Table S3.

### 2.7 Flow cytometry

The cells were seeded, 24 h later pretreated with GEM and harvested at different time points 4, 12, and 24 h after the treatment by MU380. The single‐cell suspensions were washed with PBS, fixed in 4% paraformaldehyde, permeabilized in 0.25% Triton X‐100, and stained under nonsterile conditions. For the analysis of the cell cycle, FxCycle Violet Stain diluted in PBS was used. The primary antiphospho‐histone H2A.X antibody was used to detect DNA damage. Biotin‐conjugated primary antiphospho‐histone H3 together with streptavidin PerCP‐eFluor710‐conjugated secondary antibodies were used to detect mitotic cells. Dead cells were determined by amine‐reactive LIVE/DEAD Green Cell Viability Assay (Thermofisher Scientific). For the apoptotic assay, ApoFlowEx® FITC Kit (Exbio, Prague, Czech Republic) was used according to the manufacturer's protocol. For RAD51 analysis, Alexa Fluor 488‐conjugated primary antibody was used. Dead cells were excluded by amine‐reactive LIVE/DEAD Violet Cell Viability Assay (Thermofisher Scientific). Mitochondrial membrane potential was analyzed using the tetramethylrhodamine, ethyl ester (TMRE) probe. The cells were washed with Hanks' balanced salt solution buffer and stained for 20 min in diluted TMRE solution, final concentration 0.1 μm. Cells were analyzed by BD FACSVerse (Becton Dickinson, USA, three lasers—405, 488, and 640 nm; eight detectors). Compensation values for multicolor analyses were calculated automatically in bd facssuite Software (Becton Dickinson, Franklin Lakes, NJ, USA) or flowjo (v10.0.7, Ashland, OR, USA) from single‐conjugate‐stained UltraComp eBeads (Thermofisher Scientific) or cell lines. Cell aggregates and debris were excluded from the analysis based on a dual‐parameter dot plot in which the pulse ratio (signal height/*y*‐axis vs signal area/*x*‐axis) was displayed. Material, clones, dilutions, catalog numbers, and producers are listed in Table S3.

### 2.8 Image stream analysis

Based on the protocol described above, the cells harvested 12 h after MU380 treatment were stained with primary conjugated antiphospho‐histone H2A.X and anti RAD51 antibodies, biotin‐conjugated primary antiphospho‐histone H3 with streptavidin‐PE‐Cy7‐conjugated secondary and unconjugated primary M30 CytoDEATH, together with anti‐mouse Alexa Fluor 647‐conjugated secondary antibody. Co‐staining with DAPI probe was used for the quantification of DNA content. Flow imaging was done using Amnis ImageStream Imaging Flow Cytometer (Luminex Corporation, Austin, TX, USA), with a given configuration [one charge‐coupled device (CCD) camera and six detection channels]. Material, clones, dilutions, catalog numbers, and producers are listed in Table S3.

### 2.9 Xenograft mouse experiments

Immunodeficient male mice severe combined immunodeficient (SCID) hairless outbred (SHO) (Crl:SHO‐Prkdc^scid^Hr^hr^) were from Charles River Laboratories (Wilmington, MA, USA). A total of 1 × 10^6^ of DR PDXs PC346C‐DOC and PC339‐DOC were resuspended in the 1 : 1 mix of ice‐cold PBS and Matrigel (Corning) and inoculated subcutaneously into the right flank (dorsally) of six‐week‐old male SHO mice. A week after, when tumors became palpable, nine mice per group were randomly divided into four cohorts and treated by intraperitoneal administration of either vehicle (Kolliphor ELP, Sigma‐Aldrich), GEM [150 milligram per kilogram body weight (mpk) dissolved in Kolliphor ELP, i.p. administration], SCH900776 or MU380 (25 mpk dissolved in Kolliphor ELP, i.p. administration), or combined approach as also described previously [19]. The treatment was performed in three cycles weekly (Fig. 5A). Tumor size was measured twice a week by caliper. Tumor volume was calculated using formula volume (mm^3^) = (length × height^2^)/2. Mice were euthanized with CO_2_ 4 weeks after inoculation and the tumors were surgically excised, measured, weighed *ex vivo* and frozen. All European Union Animal Welfare lines (EU Directive 2010/63/EU for animal experiments) were respected. Animal experiments were approved by the Academy of Sciences of the Czech Republic (AVCR 65/2016), supervised by the local ethical committee and performed by certified individuals (SD and KS).

### 2.10 Statistical analysis

Data from the dose–response analysis were standardized as % of control. A nonlinear regression to generate curves with four‐parameter dose–response model: $Y=\text{Bottom}+(\text{Top}-\text{Bottom})/(1+10^{({\text{LogIC}}_{50}-X)*\text{HillSlope}})$ was used to calculate half‐maximal inhibitory concentration (IC_50_) as the concentration of agonist that gave a response that was halfway between Bottom and Top. HillSlope coefficient denoted the steepness of the sigmoidal curve; the top and bottom determined plateaus in the units of the *y*‐axis. The lower and upper bound of a 95% confidence interval for IC_50_ was calculated. Heat map generation and cluster analyses were performed with Morpheus (Broad Institute, Cambridge, MA, USA). All statistical comparisons were analyzed with an unpaired *t‐*test, one‐way ANOVA with Bonferroni correction or extra sum‐of‐squares *F*‐test with Bonferroni correction for IC_50_ comparison, where various letters denote significant differences. Groups (GEM/GEM + SCH900776/GEM + MU380) with the same letter are not detectably different while groups that are detectably different have different letters. Groups can have more than one letter to reflect overlaps. If the groups have the same letter, this does not mean they are the same, just not significantly different on the appropriate level of significance.

## 3 Results

### 3.1 CHK1 inhibition by MU380 effectively sensitizes docetaxel‐resistant PCa cells to gemcitabine

**Fig. 1:**
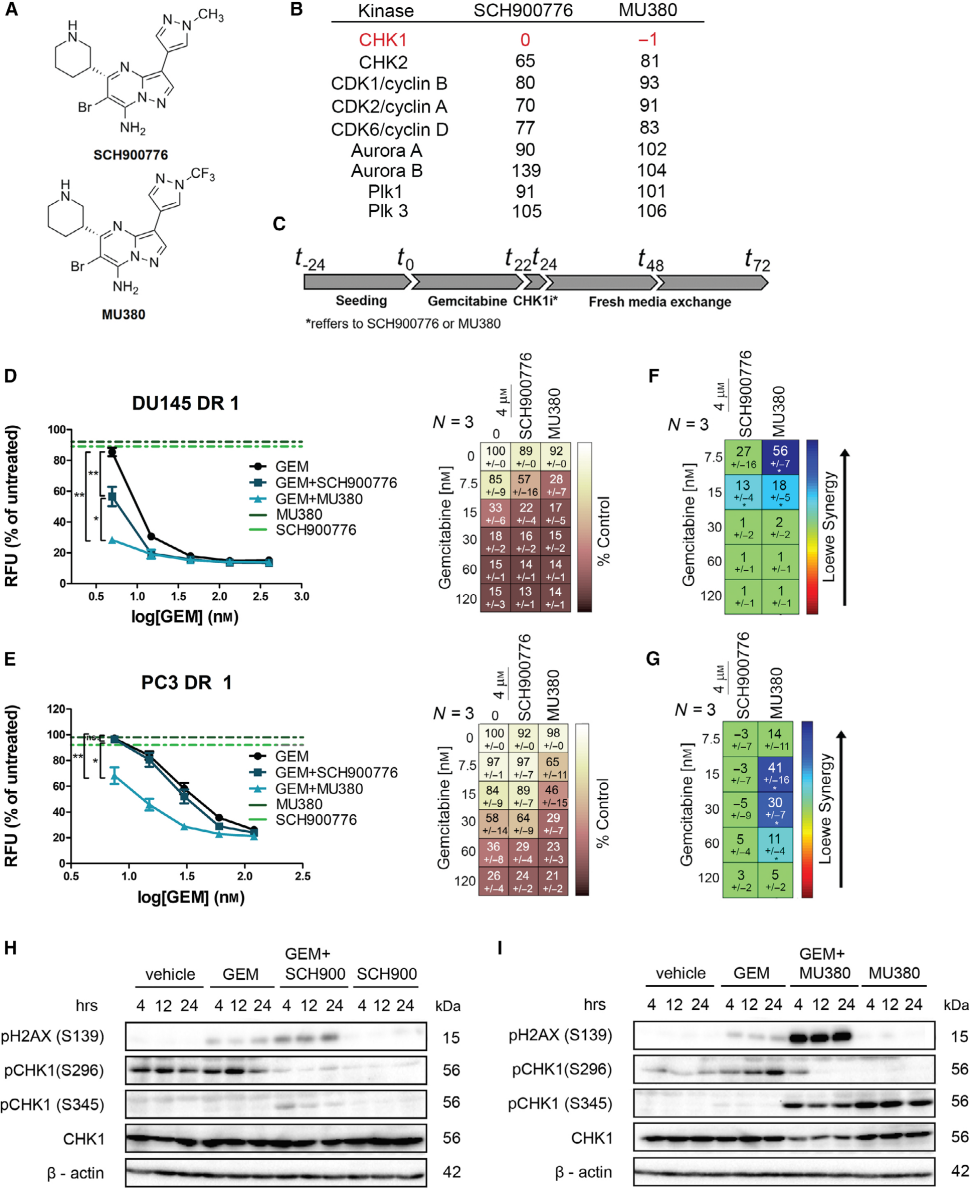
CHK1 inhibition by MU380 effectively sensitizes docetaxel‐resistant PCa cells to GEM. (A) Structure of CHK1 inhibitors SCH900776 and MU380 and (B) activity of CHK1 and other kinases involved in cell cycle regulation after *in vitro* SCH900776 or MU380 (1 µm) inhibition. (C) Timeline depicting treatment strategy. (D, F) Dose–response curves of relative viability of docetaxel‐resistant DU145 (D) and PC3 (F) cells, treated by a range of concentrations of GEM (in *x*‐axis) alone or in combination with CHK1 inhibitors (SCH900776 or MU380) and assessed by CyQUANT. The *y*‐axis indicates the percentage of viable cells relative to control (MQ water or DMSO). Data represent means ± SEM (*n* ≥ 6) from three independent biological repetitions. **P < 0.0001; *P < 0.01 by extra sum‐of‐squares *F*‐test. (E, G) Synergy score of GEM and CHK1 inhibitors on docetaxel‐resistant DU145 (E) and PC3 (G) cells analyzed using Loewe mathematical model [high synergy (dark blue), low synergy (green), antagonism (dark red)]. Data represent means ± SEM (*n* ≥ 6) from three independent biological repetitions. *P < 0.01 by Loewe mathematical model. (H, I) Western blot analysis of pH2AX, pCHK1 (S296 and S345), total CHK1 and ß‐actin as a loading control of the PC3 DR cells treated with GEM in combination with SCH900776 (H) or MU380 (I). Cells were harvested 4, 12, and 24 h after the CHK1 inhibition treatment. ns., not significant, RFU, relative fluorescence unit.

In our previous work [19], we identified MU380 as a metabolically more robust nontrivial analog of the CHK1 inhibitor SCH900776 (Fig. 1A,B). To identify the potency of MU380 to sensitize DR PCa to chemotherapy, we employed two sets of DR DU145 and PC3 cells along with their sensitive counterparts (Table S1). Considering the data from drug screenings addressing the sensitivity of all models to various chemotherapy agents with different mechanisms of action and molecular mechanism of CHK1 activation triggered by apical kinases ATM and ATR after the induction of DNA damage [26], we selected GEM as a suitable chemotherapy drug for the combined treatment (Figs [Link], [Link], [Link]). Next, we treated all DR as well as control cell lines with different concentrations of GEM for 24 h to activate DDR, followed by CHK1 inhibition by MU380 or SCH900776 for 2 h (Fig. 1C). We compared the effect of GEM monotherapy or combination with CHK1 inhibitors on proliferation and determined corresponding IC_50_ values. As shown, the combined treatment with GEM and MU380 or SCH900776 was more effective than GEM monotherapy in a majority of DR and control models, while the monotherapy by MU380 or SCH900776 had no impact on cell viability (Fig. 1D,E, Fig. S4 and Table S2). Moreover, MU380 showed significantly higher activity (reflected in the Loewe synergy score) in the sensitization of DR DU145 and PC3 cells to GEM compared to SCH900776 (Fig. 1F,G). Both CHK1 inhibitors elicited increased phosphorylation of DNA damage sensor γH2A.X on S139 (pH2AX) after GEM treatment. Again, a stronger effect was observed for combination with MU380. As expected, MU380 also effectively abrogated activation of CHK1 *via* S296 autophosphorylation while simultaneously promoting DNA damage signaling toward phosphorylation on S345 triggered by ATR (Fig. 1H,I). Taken together, these results indicate that CHK1 inhibition, especially by MU380, efficiently sensitizes DR cells to GEM leading to increased DNA damage and reduced cell viability.

### 3.2 S‐phase delay is a consequence of combined therapy‐induced cytotoxicity

**Fig. 2:**
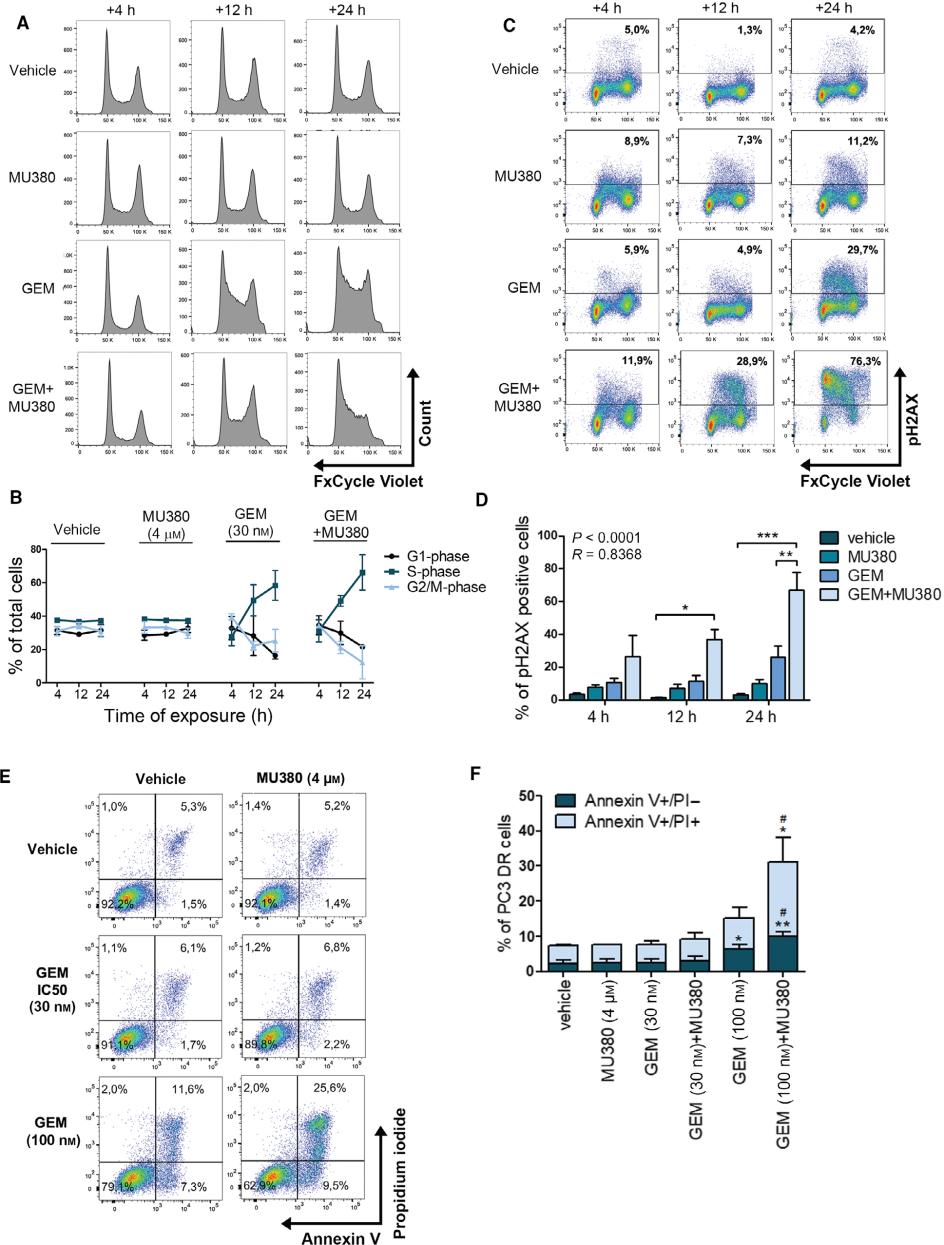
S‐phase delay as a consequence of combined therapy‐induced cytotoxicity. (A) Cell cycle analysis of PC3 DR cells using FxCycle Violet Stain. (B) Quantification of cell cycle kinetics from panel A. Data represent means ± SEM from three independent biological repetitions. (C) Analysis of pH2AX and cell cycle. (D) Quantification of DNA damage from panel C. Dead cells were excluded from the analysis based on their positivity to LIVE/DEAD stain. Data represent means ± SEM from three independent biological repetitions. *P < 0.05, **P < 0.01; ***P < 0.001 by unpaired *t*‐test. (E) Annexin V/PI‐based analysis of apoptotic cells (endpoint 24 h). (F) Quantification of Annexin/PI‐positive cells from (E). The PC3 DR cells were harvested 4, 12, and 24 h after the MU380 treatment. Data represent means ± SEM from three independent biological repetitions. *P < 0.05, **P < 0.01 compared to the vehicle by unpaired *t*‐test. ^#^ *P* < 0.05 compared to the GEM (100 nm) by unpaired *t*‐test.

Next, we aimed to elucidate the effect of CHK1 inhibition on cell cycle distribution and apoptosis. The cell cycle profile along with pH2AX and phosphorylated mitotic marker Histone H3 phosphorylated on S10 (pHH3) was investigated using quantitative flow cytometry. For this purpose, we used the PC3 DR model and previously determined IC_50_ of GEM (30 nm). We found that GEM alone increased the population of cells in S‐phase and delayed progression into G2‐phase (Fig. 2A,B). Interestingly, CHK1 inhibition by MU380 prolonged S/G2 progression and resulted in even higher accumulation of cells in S‐phase compared to GEM pretreated cells only (Fig. 2A,B). A synergistic combination of GEM and MU380 resulted in a significant increase of DNA damage, determined by pH2AX compared to vehicle and both monotherapy‐treated samples at all time points (Fig. 2C,D). Nevertheless, the combination of both drugs did not substantially alter either the population of mitotic cell death, apoptosis, or dead cells at the endpoint of 24 h (Fig. 2E,F and Fig. S5A–F), in contrast to the significantly reduced cell viability at 48 h as shown before. Usage of sublethal concentration of GEM corresponding to IC_75_ increased both early apoptosis and late apoptosis/secondary necrosis (Fig. 2E,F), indicating that higher GEM concentration was able to induce a stronger DDR response. This was further exacerbated by the significant increase of apoptosis in DU145 DR cells (Fig. S5G,H). Together, these data suggest that S‐phase delay is the consequence of the cytotoxicity induced by combined treatment of GEM and MU380, while forced mitotic cell death is not the cause of cell death in the DR PC3 DR model.

### 3.3 MU380 induces cell death in docetaxel‐resistant PCa patient‐derived xenografts *in vitro*

**Fig. 3:**
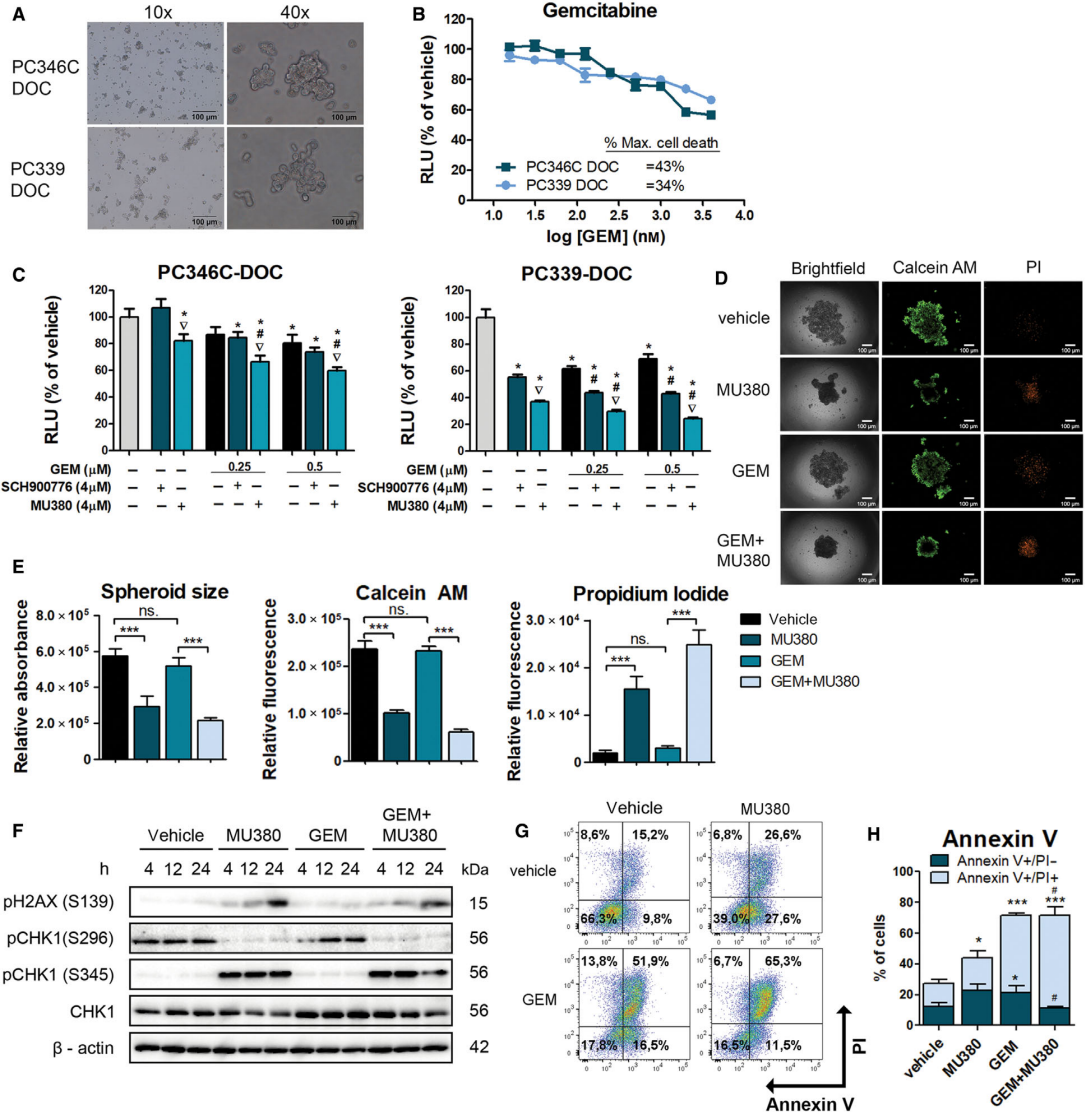
MU380 induces cell death in DR PCa PDXs *in vitro*. (A) Morphology of PC346C‐DOC and PC339‐DOC spheroids culture. Scale bar 100 μm. (B) GEM dose–response curves of relative viability of PC346C‐DOC and PC339‐DOC. Data represent means ± SEM (*n* ≥ 6) from three independent biological repetitions. (C) 3D spheroid assay, relative viability of PC346C‐DOC and PC339‐DOC spheroids treated by GEM alone (0.25 and 0.5 µm) or in combination with CHK1 inhibitors (4 µm SCH900776 or MU380). Control spheroids were treated with vehicle. The *y*‐axis refers to a percentage of viable cells relative to vehicle (MQ water or DMSO). Data represent means ± SEM (*n* ≥ 10) from two independent biological repetitions. *P < 0.05 treatment vs control; ^#^ *P* < 0.05 GEM + SCH900776 or GEM + MU380 vs GEM alone; ▽*P* < 0.05 MU380 vs SCH900776 (alone or in combination) by unpaired *t*‐test. (D, E) Representative images and quantification of spheroid size and viability determined by calcein AM/PI of PC339‐DOC spheroids treated with GEM (0.5 µm) or MU380 (4 µm) alone or their combination in the endpoint of the 3D spheroid assay. Scale bar 100 μm. Data represent means ± SEM (*n* ≥ 10) from two independent biological repetitions. ***P < 0.001; by unpaired *t*‐test. (F) Western blot analysis of pH2AX, pCHK1 (S296 and S345), total CHK1, and ß‐actin as a loading control on PC339‐DOC spheroid cell culture. (G, H) Annexin/PI‐based analysis of apoptotic cells and their quantification (H) on PC339‐DOC spheroid cell culture treated with GEM (0.5 µm) or MU380 (4 µm) alone or their combination. Data represent means ± SEM from three independent biological repetitions. *P < 0.05, ***P < 0.001 compared to the vehicle by unpaired *t*‐test. ^#^ *P* < 0.05 compared to the GEM (100 nm) by unpaired *t*‐test. RLU, relative luminescence unit.

To further examine the effect of MU380, we employed two previously established clinically relevant DR PDX models [25]. DR PDXs were established *in vivo* by serial passaging of androgen‐responsive PC346C and androgen‐independent PC339 in male athymic mice under docetaxel pressure until resistance [25]. Both models bear a wild‐type sequence of *TP53* and can be cultivated as floating 3D structures *in vitro* (Fig. 3A and Table S1). Here, we investigated the effect of GEM alone as well as in combination with SCH900776 or MU380 on cell viability *in vitro* in 3D spheroid cultures using the 3D spheroid assay. Dose–response analysis performed on floating spheroid cultures of PDXs revealed limited response to GEM alone (maximum cell death rate ~43% and ~34% in PC346C‐DOC and PC339‐DOC, respectively) in both models (Fig. 3B). Further, we selected two concentrations of GEM (0.25 and 0.5 µm) and combined its effect with 4 µm MU380 or SCH900776, in 3D single spheroid conditions. Both doses of GEM elicited similar cell response and resulted in 20% or 40% viability reduction for PC346C‐DOC or PC339‐DOC, respectively. The combinations with MU380 contributed to a significantly reduced cell viability in both DR as well as naïve models (Fig. 3C and Fig. S6). Compared to SCH900776, the MU380 monotherapy was more cytotoxic in both models (Fig. 3C). As expected, GEM (0.5 µm) alone did not strongly affect spheroid size and viability. However, the combination with MU380 resulted in a significant reduction of both spheroid size and viability (Fig. 3D,E and Fig. S6A–D). We also observed a significant regression of spheroid size in MU380 monotherapy‐treated spheroids (Fig. 3D,E). On the molecular level, CHK1 inhibition by MU380 disables autophosphorylation of CHK1 on S296 while signals DNA damage by phosphorylation of pH2AX and promotes DNA damage signaling *via* ATR‐dependent CHK1 phosphorylation on S345 (Fig. 3F). Besides, treatment by MU380 led to significantly decreased mitochondrial membrane potential (Fig. S7) and an increased number of apoptotic cells, determined by Annexin V/PI assay (Fig. 3G,H). Altogether, these results revealed unique activity and significance of MU380 as monotherapy and in combination with GEM in clinically relevant docetaxel naïve and resistant PCa models.

### 3.4 MU380‐driven premature mitosis is the major cause of patient‐derived xenograft cell death

**Fig. 4:**
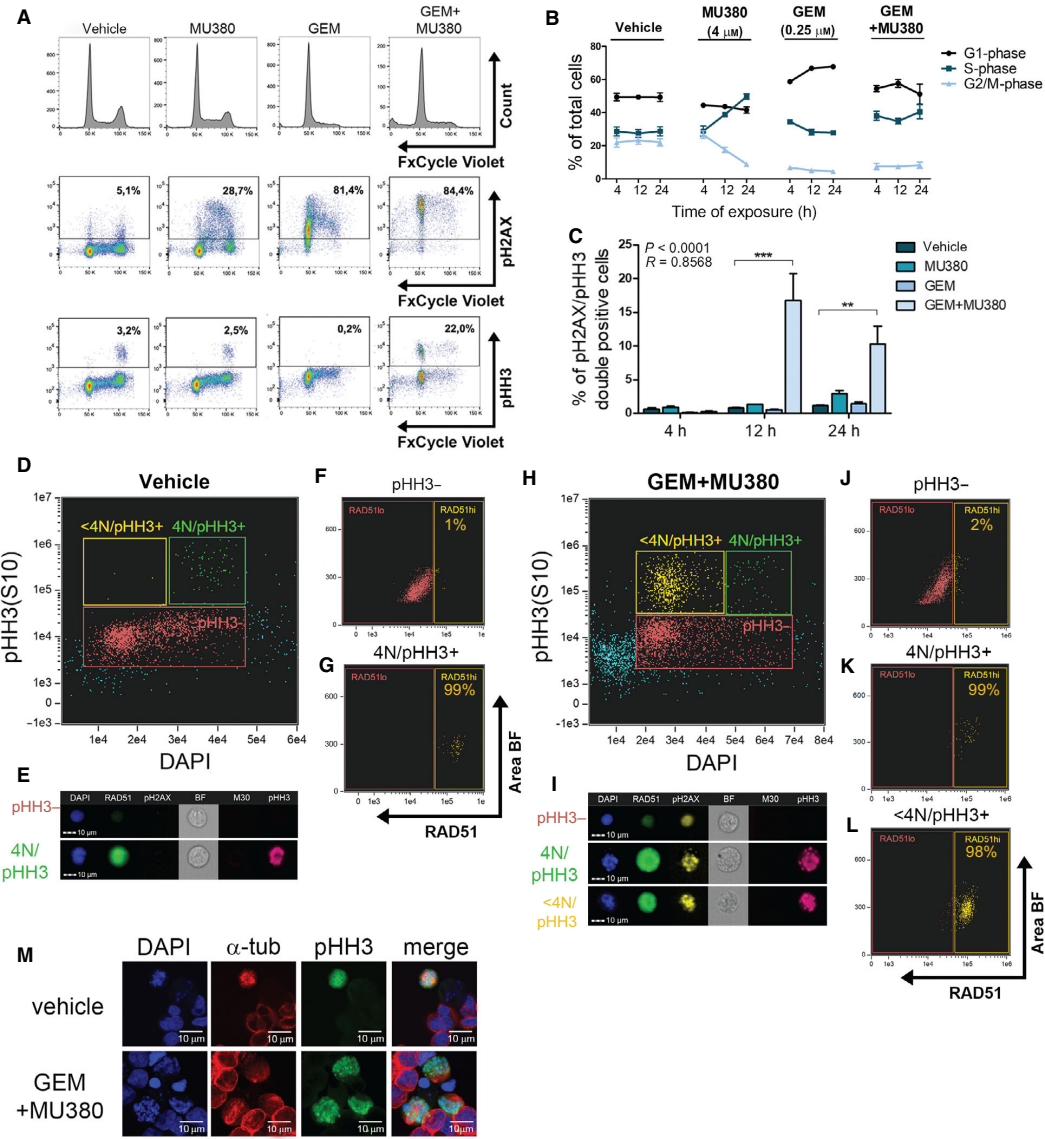
MU380‐driven premature mitosis is the major cause of PDX cell death. (A) Flow cytometry analysis of cell cycle (FxCycle Violet), DNA damage (pH2AX), and mitotic cells (pHH3) at the time point 12 h upon indicated treatment of the PC339‐DOC model. (B) Quantification of cell cycle depicted as kinetics in three time points for all treatments. Data represent means ± SEM from three independent biological repetitions. (C) Quantification of double‐positive (pH2AX and pHH3) cells. The cells were harvested 4, 12 and 24 h after the MU380 treatment. Dead cells were excluded from the analysis based on their positivity to LIVE/DEAD stain. Data represent means ± SEM from three independent biological repetitions. **P < 0.01; ***P < 0.001 by unpaired *t*‐test. Multiparametric imaging flow cytometry analysis of DNA content (DAPI), DNA repair (RAD51), DNA damage (pH2AX), apoptosis (M30), and mitosis (pHH3) 12 h after MU380 treatment. Cell cycle distribution vs cells in mitosis in vehicle‐ (D) or GEM + MU30‐treated (H) cells. RAD51 positivity in the pHH3 negative (F) or positive (G) subpopulations in the vehicle‐treated and pHH3 negative (J), pHH3 positive from G2‐phase (K), or pHH3 positive from G1 and S‐phase (L) in the GEM + MU380‐treated PC339‐DOC cells. Representative images of vehicle‐treated (E) or GEM + MU380‐treated (I) cells from pregated subpopulations (D, H). (M) Microscopic analysis of DAPI, a‐tubulin, and pHH3. The cells were harvested 12 h after the MU380 treatment. Scale bar 10 µm.

To elucidate the mechanism of how CHK1 inhibition leads to the reduction of cell viability, we analyzed the level of DNA damage simultaneously with DNA content, mitotic marker, and viability using multicolor flow cytometry. Pretreatment of PC339‐DOC cells by 0.25 μm GEM for 24 h induced G1‐arrest associated with a strong increase of DNA damage compared to untreated cells (Fig. 4A). Noticeably, these cells were not able to repair DNA and to proceed through the cell cycle as they started to die from G1‐phase in a time‐dependent manner (Fig. S8A). Conversely, the addition of 4 μm MU380 to GEM pretreated cells led to G1‐checkpoint abrogation and progression to S‐phase at 24 h (Fig. 4A,B), further confirming the effect of CHK1 inhibition. It also led to a massive increase of pH2AX‐ and pHH3‐double‐positive cells from G1‐phase within 12 h (Fig. 4C and Fig. S8B–F), indicating that these cells entered premature mitosis despite GEM‐induced DNA damage (Fig. 4C and Fig. S8B). Subsequently, this resulted in a robust increase in cell death after an extra 12‐ and 24‐hr period compared to the cells treated with GEM alone (Fig. S8G,H). Moreover, a single treatment of 4 μm MU380 induced S‐phase arrest within 12 h with increased DNA damage and the number of dead cells in comparison with untreated cells. Since the activation of DNA repair mechanisms is CHK1 dependent, the levels of RAD51 (a signaling protein downstream of CHK1 that assists with the repair of damaged DNA *via* homologous recombination) were analyzed using flow cytometry. We observed a significant elevation of the RAD51 signal after treatment with GEM, which was accompanied by a sharp decrease to the basal level upon the treatment with MU380 (Fig. S9A–C). Interestingly, a strong increase in the subpopulation of RAD51 high cells was observed upon the application of the combined therapy. This subpopulation of cells was also positive for pHH3 as well as pH2AX but not for the marker of apoptosis M30, which is a product of cytokeratin 18 cleavage (Fig. 4D–L). Therefore, we investigated whether the potential mechanism of cell death might be associated with mitotic catastrophe. We observed that combined treatment resulted in an enhanced number of pHH3‐positive mitotic cells with multinuclearization and disrupted cell division (Fig. 4M and Fig. S9D) as hallmarks of mitotic catastrophe. In summary, CHK1 inhibition by MU380 results in the bypass of the GEM‐induced G1‐arrest leading to premature mitosis, which is the main cause of cell death in this DR PCa model.

### 3.5 MU380 effectively inhibits tumor growth in docetaxel‐resistant xenograft models

**Fig. 5:**
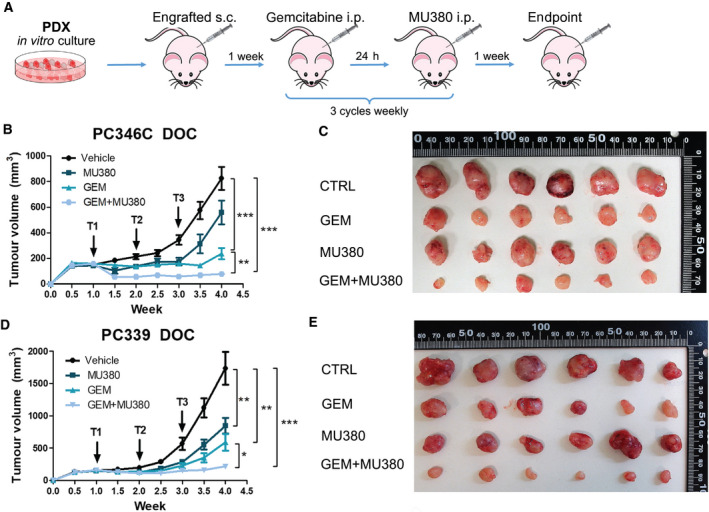
MU380 effectivity in DR PC346C and PC339 *in vivo* xenograft models. (A) Scheme depicting the *in vivo* experiment. (B, D) Plots representing PC346C‐DOC (B) or PC339‐DOC (D) xenograft tumor volume in the mice treated with GEM or MU380 alone or their combination on days 7, 14, and 21. Data represent tumor volume means ± SEM (*n* = 9). *P < 0.05, **P < 0.01, ***P < 0.001 by repeated‐measures ANOVA. (C, E) Representative image of tumor size from differently treated groups of PC346C‐DOC (C) or PC339‐DOC (E) tumor‐bearing mice. Data represent tumor volume means ± SEM (*n* = 9). s.c., subcutaneously; i.p. intraperitoneally.

Finally, the antitumor effectivity of MU380 alone or in combination with GEM was investigated *in vivo* using PC346C‐DOC or PC339‐DOC PDXs. Immunodeficient male SHO mice were subcutaneously injected with PC346C‐DOC or PC339‐DOC cells and left one week to develop xenograft tumors. Tumor‐bearing mice were treated with three cycles of GEM (150 mpk) followed by a bolus of MU380 (25 mpk) after 24 h (combined therapy), or with one bolus of GEM or MU380 or vehicle at 1, 2, and 3 weeks after inoculation. Tumor size was measured twice a week (Fig. 5A). We observed a significant reduction of tumor weight and strong tumor growth inhibition in both PC339‐DOC and PC346C‐DOC models (87% and 90%, respectively) in the combined therapy group, which was significantly more pronounced than either monotherapy alone (Fig. 5B–E and Fig. S10A–C). Notably, MU380 was effective also as monotherapy in the reduction of tumor size and weight of PC339‐DOC and PC346C‐DOC xenograft models (Fig. 5B–E, Fig. S10A–C). The treatments did not significantly affect mice body weights (Fig. S10D). Collectively, these data suggest that inhibition of CHK1 by MU380 significantly potentiates the *in vivo* efficacy of GEM and thus represents a promising approach for the therapy of advanced DR PCa.

## 4 Discussion

Despite a better understanding of molecular mechanisms and advances in the therapy of PCa, treatment options for lethal, advanced mCRPC are still rather limited. Small molecules represent an attractive and promising group of agents that specifically target protein kinases employed in different signaling pathways. Among these, DDR pathways have been considered as suitable therapeutic targets alone, or in combination with chemotherapy (Fig. S11A). Specifically, many studies have been focusing on targeting CHK1 (mostly in GEM pretreated cells) as a promising approach to eradicate different types of cancer [27, 28, 29, 30, 31] because of its unique and crucial role in the maintenance of genomic integrity [32]. However, the effectivity and the exact mechanism of this treatment strategy have not been investigated in aggressive, chemoresistant subtypes of mCRPC. Herein, we comprehensively investigate the ability of the novel CHK1 inhibitor MU380 [19], a nontrivial analog of SCH900776 [33], to potentiate the efficacy of GEM in DR PCa models (Fig. S11A).

One of the most frequently asked questions in studies concerning inhibition of protein kinases employed in DDR or cell cycle regulation, particularly CHK1, is whether the p53 function or dysfunction affects therapeutic efficacy. A recent study of patient samples from metastatic soft‐tissue sarcomas identified *TP53* mutation as a crucial determinant of CHK1 inhibition effectivity alone or in combination with GEM [34]. Nevertheless, in our previous study, we observed no difference in the sensitization of the *TP53*‐wt vs *TP53*‐mut cancer model HTC116 to chemotherapy [19]. Similarly, CHK1 inhibition was found to augment the effect of nucleoside analog fludarabine in incurable chronic lymphocytic leukemia irrespective of *TP53* status [21, 35]. This is consistent with our current data demonstrating that both CHK1 inhibitors, SCH900776 and particularly MU380, effectively sensitized DR *TP53‐null* PC3 and *TP53^+/−^* DU145 cell lines as well as DR *TP53‐wt* PC346C and PC339 *in vitro* spheroid cultures to GEM in a time‐dependent manner. Irrespective of the significantly higher efficacy of MU380 compared to the clinical candidate SCH900776, the effects of both inhibitors were associated with specific inhibition of CHK1 autophosphorylation on S296 and induction of DDR *via* ATR‐dependent phosphorylation of CHK1 on S345. Furthermore, the level of the DNA damage marker pH2AX was highest in cases of combined treatments, suggesting replication stress as a consequence of GEM sensitization.

Given the role of CHK1 in the regulation of intra S‐ and G2/M‐checkpoints [36], most studies attributed CHK1 inhibition‐induced sensitization of GEM‐treated cells to cell cycle arrest abrogation associated with premature mitotic entry [37]. Nevertheless, the response of cells to combined therapy seems to be more complex and depends on the degree of induced genotoxicity. In previous studies, lower concentrations of GEM resulted in prolongation of DNA synthesis with modest S‐phase arrest [38], ultimately enabling mitotic entry. Koh et al. demonstrated that CHK1 inhibition of GEM pretreated cells results in a prolonged delay in the progression through S‐phase accompanied with enhanced DNA damage [29] (Fig. S11B). In contrast, higher doses of GEM induced an irreversible proliferation block and synchronization of cells in G1‐phase arrest [39], which resulted in forced, premature mitosis upon CHK1 inhibition [29, 40] (Fig. S11C). This is consistent with our data, wherein the PC3 DR cell line, a synergistic combination (in terms of cytotoxicity) of GEM (30 nm) with MU380 engages cells in disordered prolonged replication and then commits them to elimination processes beyond mitosis. On the other hand, a high dose of GEM (0.5 µm) in the PC339‐DOC model induced uniform and permanent arrest in the G1 phase with time‐dependent G1‐phase mitotic entry upon the MU380 treatment, resulting in mitotic catastrophe and cell death. This suggests that forced mitosis is not a unique mechanism responsible for the sensitization of GEM by CHK1 inhibition. Other mechanisms including disruption of HR *via* RAD51 inhibition [41] or destabilization of DNA replication [29] are likely to be involved, depending on the extent of DNA damage induced by GEM dose.

Upon CHK1 inhibition, the cytotoxic effects are attributed predominantly to increased rates of DNA replication [19, 29]. Considering CHK1 as a guard of normal S‐phase progression [42], loss of the CHK1‐dependent DNA damage checkpoint might result in a replicative catastrophe and unavoidable cell death [43], suggestive for the potential use of CHK1 inhibition also as monotherapy. Numerous studies reported CHK1 inhibition as a promising monotherapy option to eradicate different types of malignancies [44, 45, 46] and bypass chemoresistance [47]. This approach is investigated also in clinical trials [48, 49]. In consistence with the previous study [21], we observed a significant decrease in the viability of the MU380‐monotherapy‐treated PC346C‐DOC and PC339‐DOC spheroid cultures *in vitro*. This fact might be attributed to the functional status of the AR. The AR was shown to be mechanistically linked to DDR, more precisely to the TopBP1‐ATR‐CHK1 axis, acting as an upstream molecule which upregulates the expression of specific DDR and DNA repair genes associated with metastasis, castration resistance, and reduced overall survival of PCa patients [18, 19, 50, 51]. Karanika *et al*. proposed the synergy between CHK1 and AR/CDC6 inhibition as an effective strategy to induce DNA damage and apoptosis, leading to effective treatment of mCRPC [17, 19]. These findings correlate with our data from both *in vitro* and *in vivo* experiments showing higher effectivity of the MU380 monotherapy in all AR‐independent models compared to the AR‐responsive PC346C model. Thus, single‐agent CHK1 inhibition by MU380 may serve as an important and promising therapeutic strategy for mCRPC patients.

Observations from an *in vitro* PCa model have shown that GEM acts in an antiproliferative as well as inhibitory colony formation manner [52]. In the clinical view, GEM may not be appropriate as first‐line therapy in PCa due to hematotoxicity and discrepancy between the PSA response and the disease control rate [53]. To increase the probability of clinical success, an agent that causes DNA damage, oxaliplatin, was added to GEM in a clinical trial in patients with PCa after failure of chemotherapy [54]. Based on the PSA response rate of 55% and radiologic response rate of 82%, one could expect that advanced PCa treatment may build upon combined therapy with GEM. Increased sensitivity to GEM was supposed to be associated with the upregulation of ABCB1, which in contrast played a pivotal role in the development of docetaxel resistance [55]. Even though it is still not clear whether docetaxel‐resistant patients could benefit from GEM therapy, a recent study has demonstrated that GEM may be beneficial to effectively induce tumor regression in the DR CRPC model.

## 5 Conclusion

The results of our experimental study provide evidence that targeting CHK1 by MU380 can be used to significantly improve the effectiveness of the clinically used drug GEM, which supports the previous statements (Fig. S11). Consequently, this combination might allow for using lower doses of GEM, thereby reducing the risk of major side effects [53]. This strategy might be potentially also applicable to chemotherapy‐naïve patients to avoid high toxicity and side effects of chemotherapy and prevent the development of docetaxel resistance. Altogether, these data provide a preclinical rationale for the use of the CHK1 inhibitor MU380 in a clinical setting for the therapy of incurable mCRPC.

## Conflict of interest

The authors declare no conflict of interest.

## Author contributions

SD performed experiments and analyzed the data, interpreted the data, and wrote and reviewed the manuscript. PK and KP performed organic synthesis and profiling of CHK1 inhibitors. WMvW established PDX models. RF and TS helped with *in vitro* experiments and analyses. DB and JČ helped with image cytometry measurements. MP and WMvW established chemoresistant cell lines. AH, WMvW, WRW, ZC, LK, KP interpreted the data, wrote, and reviewed the manuscript. KS conceptualized and designed the study, interpreted the data, wrote and reviewed the manuscript, and supervised the study. All authors read and approved the final version of this manuscript.

## Supporting information


**Fig. S1.** Heatmap and clustering analysis of drug response.

**Fig. S2.** A dose‐response analysis of chemotherapy sensitivity in DU145 cells.

**Fig. S3.** A dose‐response analysis of chemotherapy sensitivity in PC3 cells.

**Fig. S4.** The inhibition of CHK1 sensitizes PCa cells to GEM.

**Fig. S5.** S‐phase delay as a consequence of combined therapy‐induced cytotoxicity.

**Fig. S6.** MU380 induces cell death in PCa PDXs *in vitro*.

**Fig. S7.** MU380 induces a decrease in mitochondrial potential in PCa PDXs *in vitro*.

**Fig. S8.** MU380‐driven premature mitosis is the major cause of PDX cell death.

**Fig. S9.** Induction of premature mitosis after combined treatment.

**Fig. S10.** MU380 effectivity in docetaxel‐resistant PC346C and PC339 *in vivo* xenograft models.

**Fig. S11.** CHK1 inhibition potentiates the cytotoxic effect of gemcitabine.

**Table S1.** Characteristics of PCa models.

**Table S2.** IC_50_ values corresponding to dose‐response analysis from Fig. 1 and Fig. S4.

**Table S3.** Overview of antibodies and other reagents used for immunoblotting, flow cytometry, imaging flow cytometry and immunostaining.
