# Supplementary material for: The CHK1 inhibitor MU380 significantly increases the sensitivity of human docetaxel‐resistant prostate cancer cells to gemcitabine through the induction of mitotic catastrophe
Source: Mol Oncol. 2020 Jul 16;14(10):2487–503. doi: 10.1002/1878-0261.12756 (PMC7530791; doi:10.1002/1878-0261.12756)
Supplement: Supplementary file 1 — Fig. S1. Heatmap and clustering analysis of drug response. [file MOL2-14-2487-s001.pdf]

Figure S1

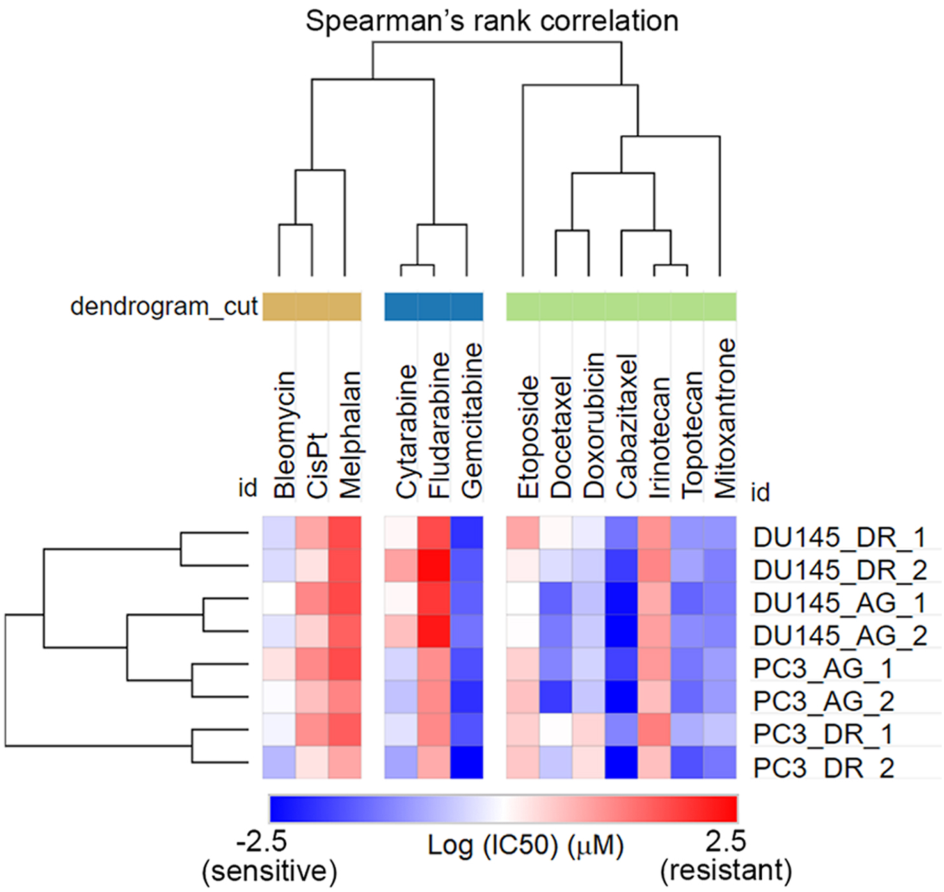

Figure S1: Heatmap and clustering analysis of drug response. Heatmap and clustering analysis (Spearman rank correlation) interpreting sensitivity (from -2.5 (dark blue) to 2.5 (dark red)) to various chemotherapy drugs of IC50 values (treatment 48 hours) assessed by proliferation assay CyQUANT.
