# Supplementary material for: The CHK1 inhibitor MU380 significantly increases the sensitivity of human docetaxel‐resistant prostate cancer cells to gemcitabine through the induction of mitotic catastrophe
Source: Mol Oncol. 2020 Jul 16;14(10):2487–503. doi: 10.1002/1878-0261.12756 (PMC7530791; doi:10.1002/1878-0261.12756)
Supplement: Supplementary file 3 — Fig. S3. A dose‐response analysis of chemotherapy sensitivity in PC3 cells. [file MOL2-14-2487-s003.pdf]

Figure S3

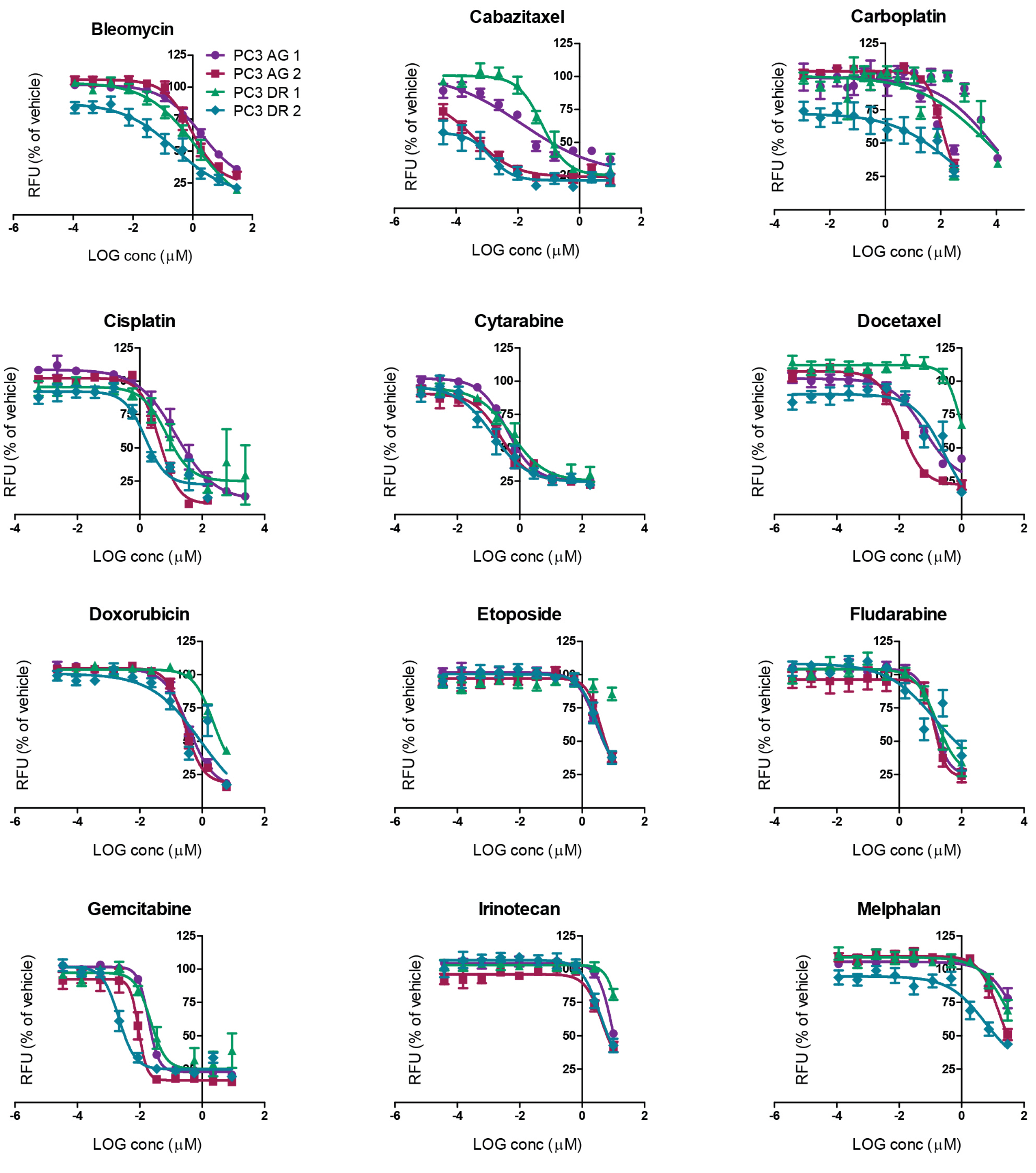

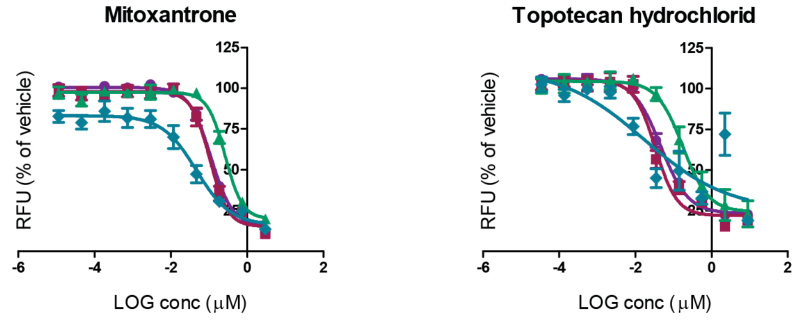

Figure S3: A dose-response analysis of chemotherapy sensitivity in PC3 cells. Dose-response curves of relative viability of PC3 cells, treated by various chemotherapy drugs for 24hrs assessed by proliferation assay CyQUANT. The X-axis indicates the log concentration range of appropriate drugs. Data represent means ± SEM (n=9) from three independent biological repetitions.
