# Supplementary material for: The CHK1 inhibitor MU380 significantly increases the sensitivity of human docetaxel‐resistant prostate cancer cells to gemcitabine through the induction of mitotic catastrophe
Source: Mol Oncol. 2020 Jul 16;14(10):2487–503. doi: 10.1002/1878-0261.12756 (PMC7530791; doi:10.1002/1878-0261.12756)
Supplement: Supplementary file 4 — Fig. S4. The inhibition of CHK1 sensitizes PCa cells to GEM. [file MOL2-14-2487-s004.pdf]

Figure S4

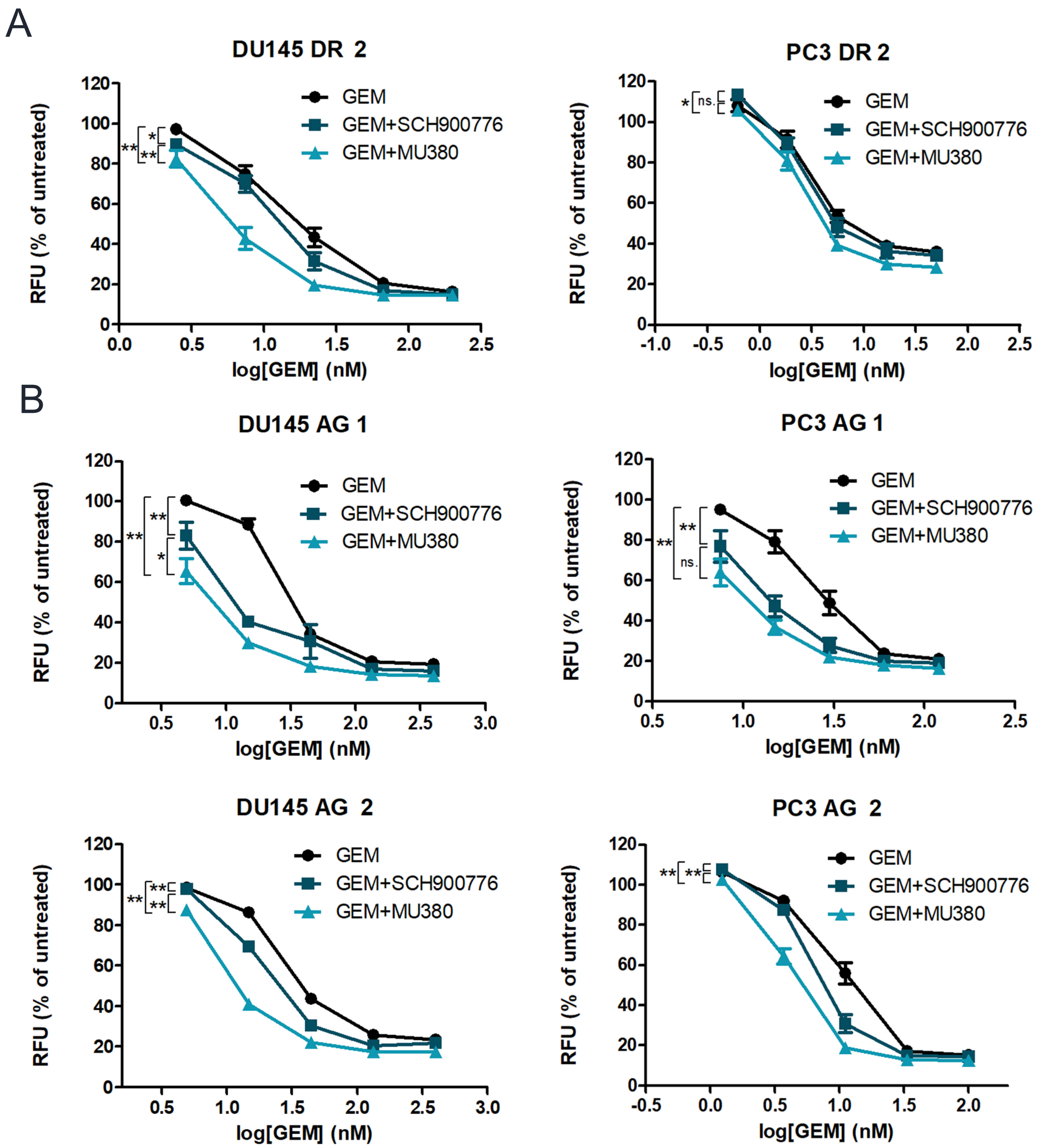

Figure S4: The inhibition of CHK1 sensitizes PCa cells to gemcitabine. A, B Dose-response curves of relative viability of DU145 and PC3 cells, treated by a concentration range of gemcitabine (in the x-axis) alone or in combination with CHK1 inhibitors (SCH900776, or MU380) assessed by the proliferation assay CyQUANT. The y-axis indicates the percentage of viable cells relative to control (water or DMSO). Data represent means ± SEM (n≥6) from three independent biological repetitions. **, P<0.0001; *, P<0.01 by unpaired t-test. GEM, gemcitabine; RFU, relative fluorescence unit; ns., not significant.
