# Supplementary material for: The CHK1 inhibitor MU380 significantly increases the sensitivity of human docetaxel‐resistant prostate cancer cells to gemcitabine through the induction of mitotic catastrophe
Source: Mol Oncol. 2020 Jul 16;14(10):2487–503. doi: 10.1002/1878-0261.12756 (PMC7530791; doi:10.1002/1878-0261.12756)
Supplement: Supplementary file 5 — Fig. S5. S‐phase delay as a consequence of combined therapy‐induced cytotoxicity. [file MOL2-14-2487-s005.pdf]

Figure S5

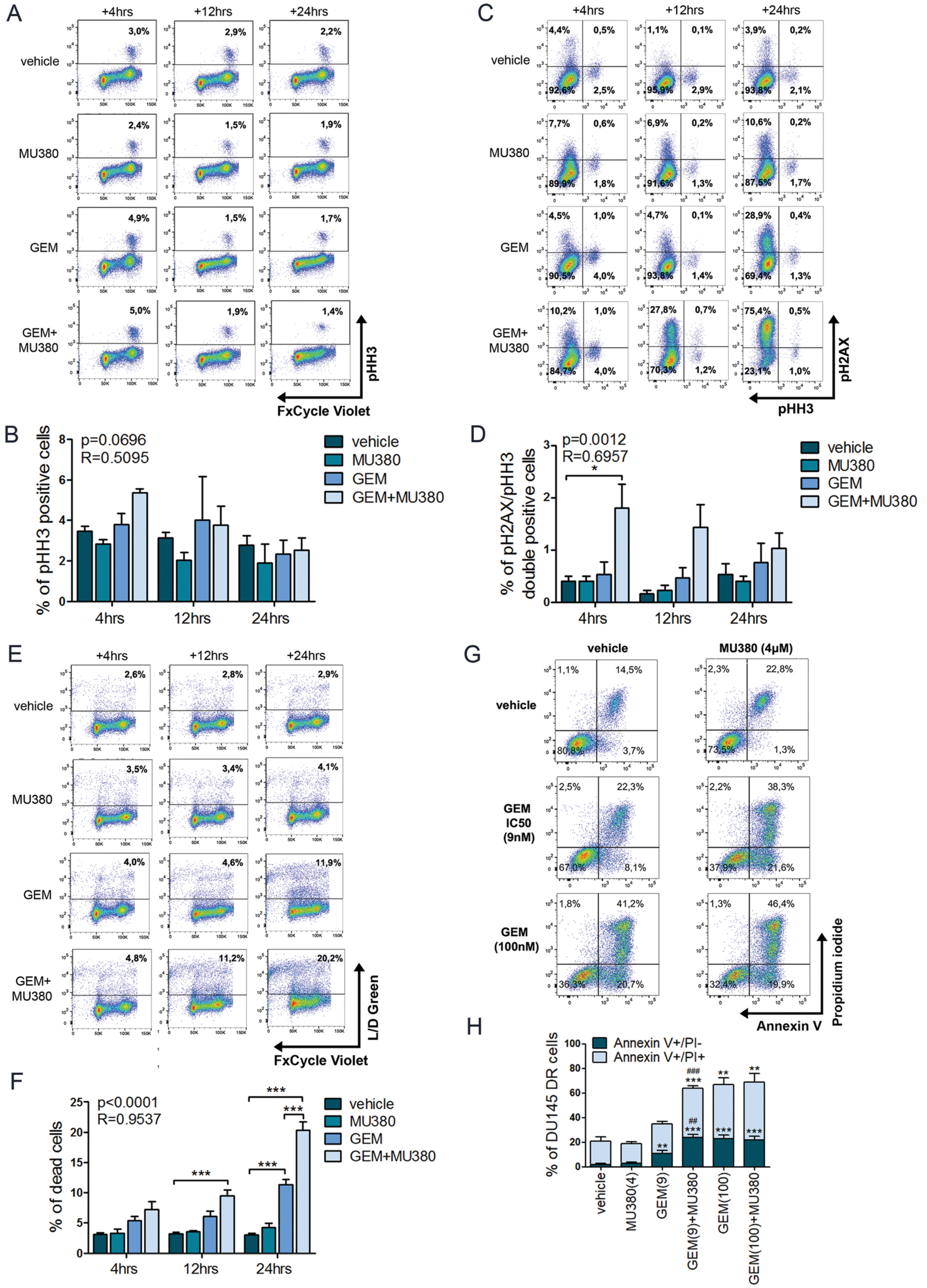

Figure S5: S-phase delay as a consequence of combined therapy-induced cytotoxicity. Multicolor flow cytometry analysis on PC3 DR cells. Dual parameter dot plot illustration of A, mitotic cells based on pHH3 positivity against cell cycle distribution. B, Quantification of mitotic cells. C, Dual-parameter dot plot (pH2AX vs. pHH3) visualization of double-positive cells. D, Quantification of double-positive (pH2AX/pHH3) cells. The cells were harvested at the time points 4, 12 and 24 hrs after the MU380 treatment. During the measurement, dead cells were excluded from the analysis based on their positivity to LIVE/DEAD stain. E, Analysis of dead cells by LIVE/DEAD Green stain. F, Quantification of dead cells. G, Annexin/PI based analysis of apoptotic cells (24 hrs). H, Quantification of Annexin/PI positive cells.
