# Supplementary material for: The CHK1 inhibitor MU380 significantly increases the sensitivity of human docetaxel‐resistant prostate cancer cells to gemcitabine through the induction of mitotic catastrophe
Source: Mol Oncol. 2020 Jul 16;14(10):2487–503. doi: 10.1002/1878-0261.12756 (PMC7530791; doi:10.1002/1878-0261.12756)
Supplement: Supplementary file 6 — Fig. S6. MU380 induces cell death in PCa PDXs in vitro. [file MOL2-14-2487-s006.pdf]

Figure S6

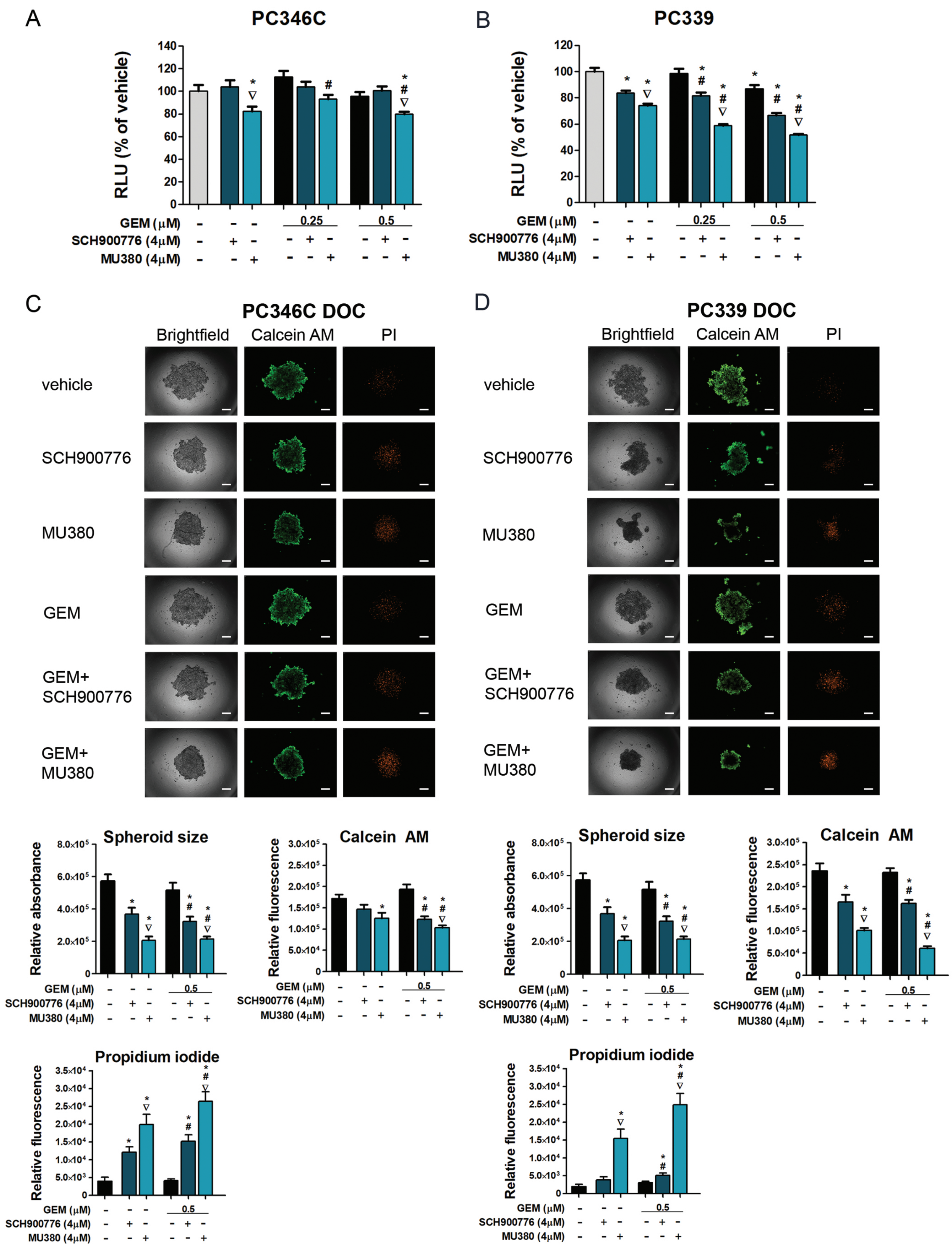

Figure S6: MU380 induces cell death in PCa patient-derived xenografts in vitro. A, B, 3D spheroid assay, relative viability of PC346C and PC339 spheroids treated by gemcitabine alone or in combination with CHK1 inhibitors SCH900776 or MU380. Two concentrations of gemcitabine (0.25 µM and 0.5 µM) were used. Control spheroids were treated with vehicle. The y-axis refers to a percentage of viable cells relative to vehicle (water or DMSO). C, D, Representative images and quantification of control PC346C and PC339 spheroid size and viability determined by Calcein AM/Propidium iodide in the endpoint of the 3D spheroid assay. Scale bar 100 µm. Data represent means ± SEM (n≥ 10) from two independent biological repetitions. *, P<0.05 treatment vs control; #, P<0.05 GEM+SCH900776 or GEM+MU380 vs GEM alone; ▼, P<0.05 MU380 vs SCH900776 (alone or in combination) by unpaired t-test. DOC, docetaxel-resistant; GEM, gemcitabine; RLU, relative luminescence unit.
