# Supplementary material for: The CHK1 inhibitor MU380 significantly increases the sensitivity of human docetaxel‐resistant prostate cancer cells to gemcitabine through the induction of mitotic catastrophe
Source: Mol Oncol. 2020 Jul 16;14(10):2487–503. doi: 10.1002/1878-0261.12756 (PMC7530791; doi:10.1002/1878-0261.12756)
Supplement: Supplementary file 7 — Fig. S7. MU380 induces a decrease in mitochondrial potential in PCa PDXs in vitro. [file MOL2-14-2487-s007.pdf]

Figure S7

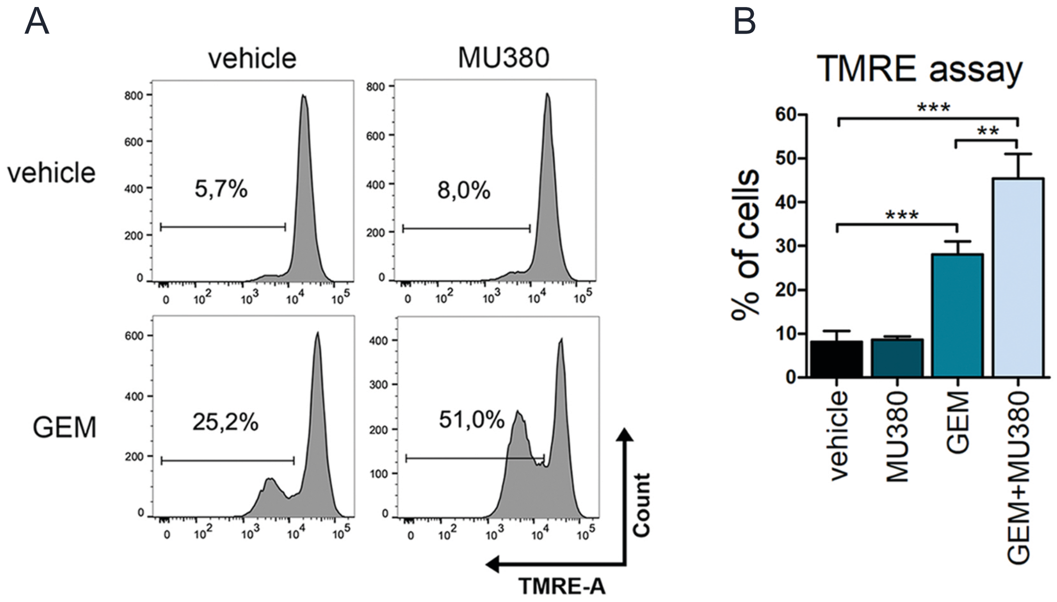

Figure S7: MU380 induces decrease in mitochondrial potential in PCa patient-derived xenografts in vitro. A, Analysis and B, Quantification of mitochondrial membrane potential using TMRE probe in PC339-DOC model treated with vehicle, gemcitabine (0.5 μM), MU380 (4 μM) or their combination at the time point 12 hrs after the MU380 treatment.
