# Supplementary material for: The CHK1 inhibitor MU380 significantly increases the sensitivity of human docetaxel‐resistant prostate cancer cells to gemcitabine through the induction of mitotic catastrophe
Source: Mol Oncol. 2020 Jul 16;14(10):2487–503. doi: 10.1002/1878-0261.12756 (PMC7530791; doi:10.1002/1878-0261.12756)
Supplement: Supplementary file 8 — Fig. S8. MU380‐driven premature mitosis is the major cause of PDX cell death. [file MOL2-14-2487-s008.pdf]

Figure S8

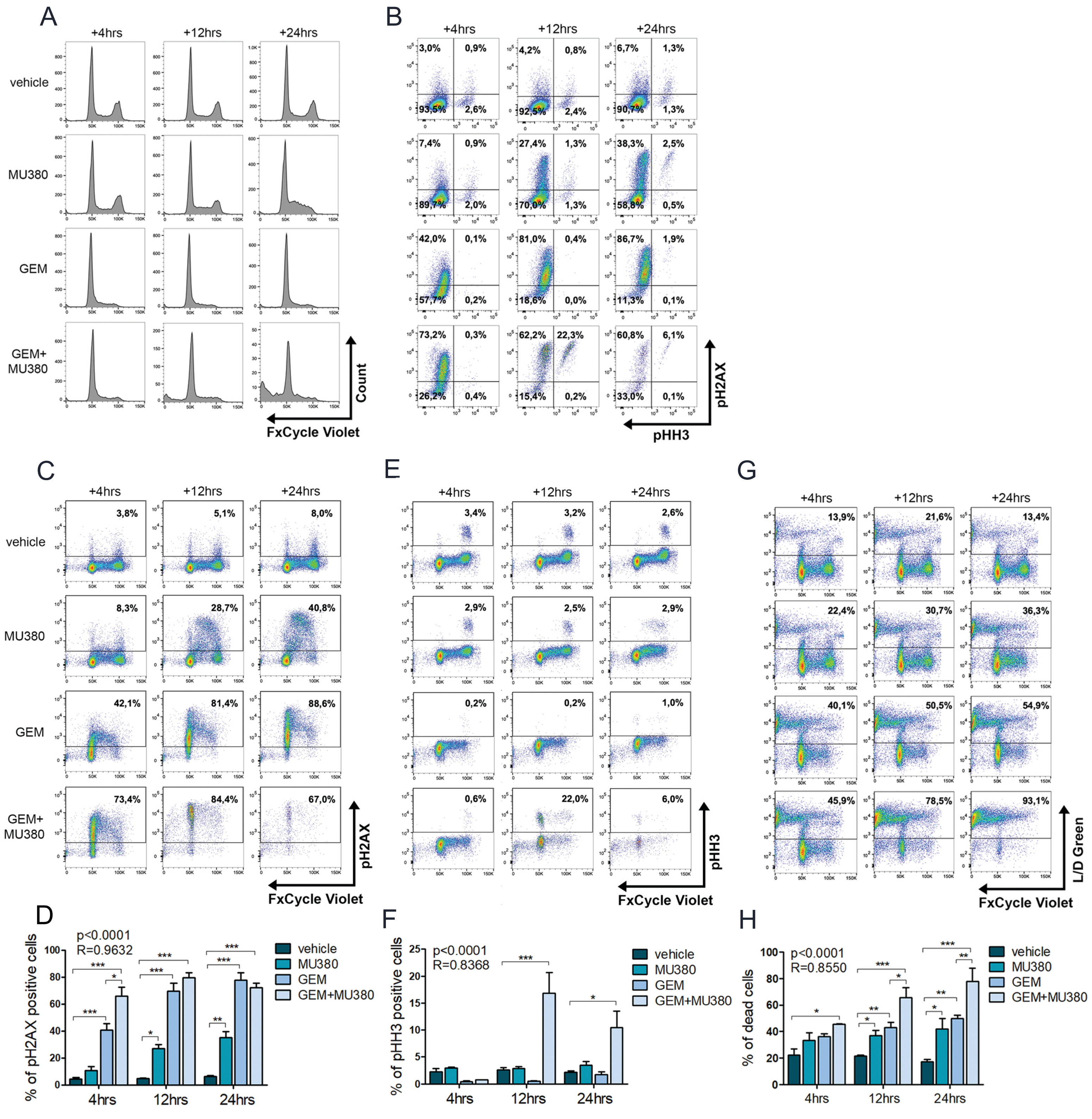

Figure S8: MU380-driven premature mitosis is the major cause of patient-derived xenograft cell death. A, Cell cycle analysis. Dual-parameter dot plot visualizations of B, pHH3 vs. pH2AX, C, D, Cell cycle vs. pH2AX plus quantification, E, F, Cell cycle vs. pHH3 plus quantification and G, H, Cell cycle vs. dead cells plus quantification The cells were harvested at the time points 4, 12 and 24hrs after the MU380 treatment.
