# Supplementary material for: The CHK1 inhibitor MU380 significantly increases the sensitivity of human docetaxel‐resistant prostate cancer cells to gemcitabine through the induction of mitotic catastrophe
Source: Mol Oncol. 2020 Jul 16;14(10):2487–503. doi: 10.1002/1878-0261.12756 (PMC7530791; doi:10.1002/1878-0261.12756)
Supplement: Supplementary file 9 — Fig. S9. Induction of premature mitosis after combined treatment. [file MOL2-14-2487-s009.pdf]

Figure S9

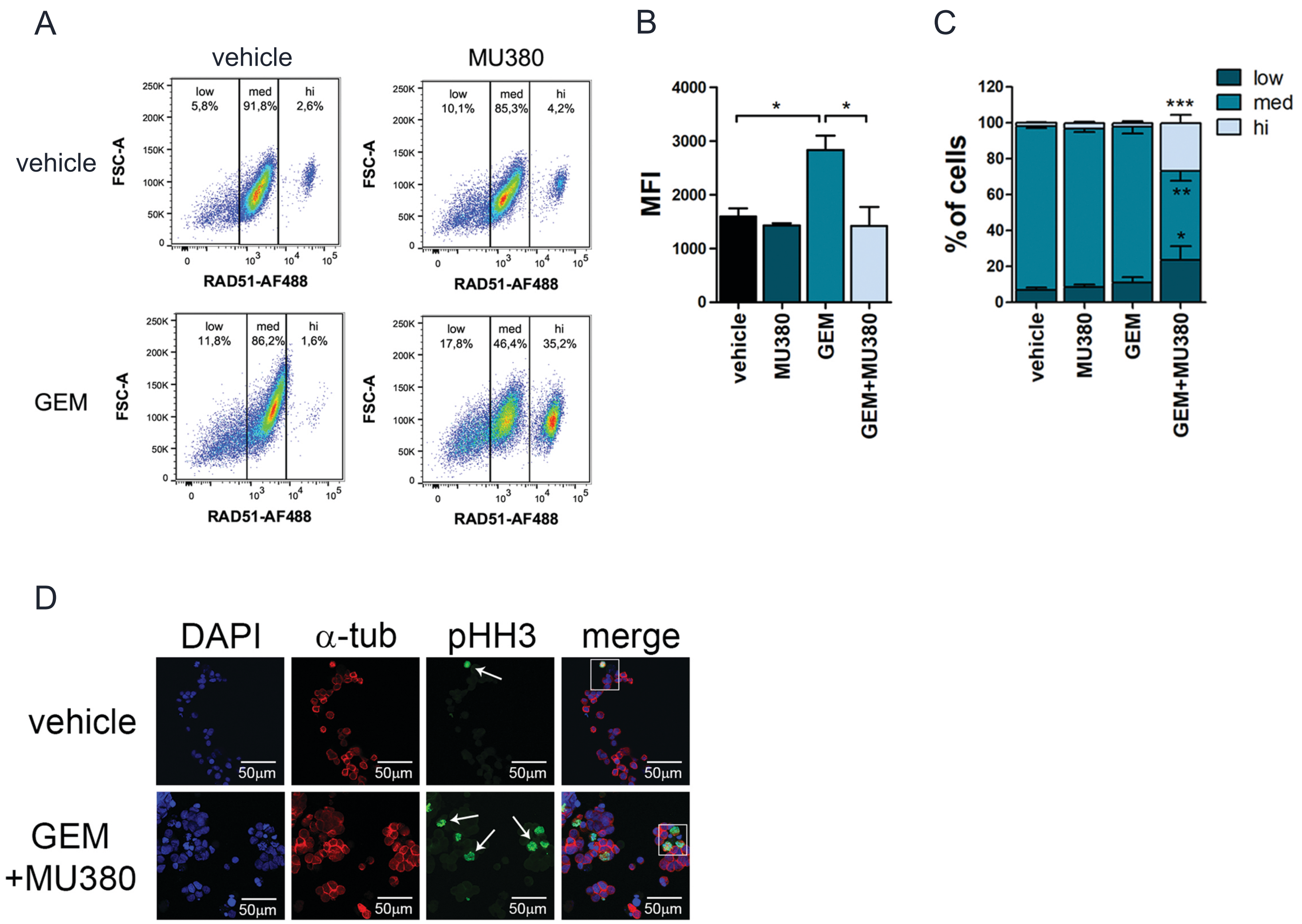

Figure S9: Induction of premature mitosis after combined treatment. A, RAD51 expression in PC339-DOC cells after different treatments. B, Quantification of MFI from three independent biological repetitions. C, Quantification of % positivity of lo/me/hi RAD51 subpopulations. D, Microscopy analysis of DAPI, a-tubulin, and pHH3. The cells were harvested at the time point 12 hrs after the MU380 treatment. Scale bar: 50 µm.
