# Supplementary material for: The CHK1 inhibitor MU380 significantly increases the sensitivity of human docetaxel‐resistant prostate cancer cells to gemcitabine through the induction of mitotic catastrophe
Source: Mol Oncol. 2020 Jul 16;14(10):2487–503. doi: 10.1002/1878-0261.12756 (PMC7530791; doi:10.1002/1878-0261.12756)
Supplement: Supplementary file 10 — Fig. S10. MU380 effectivity in docetaxel‐resistant PC346C and PC339 in vivo xenograft models. [file MOL2-14-2487-s010.pdf]

# Figure S10

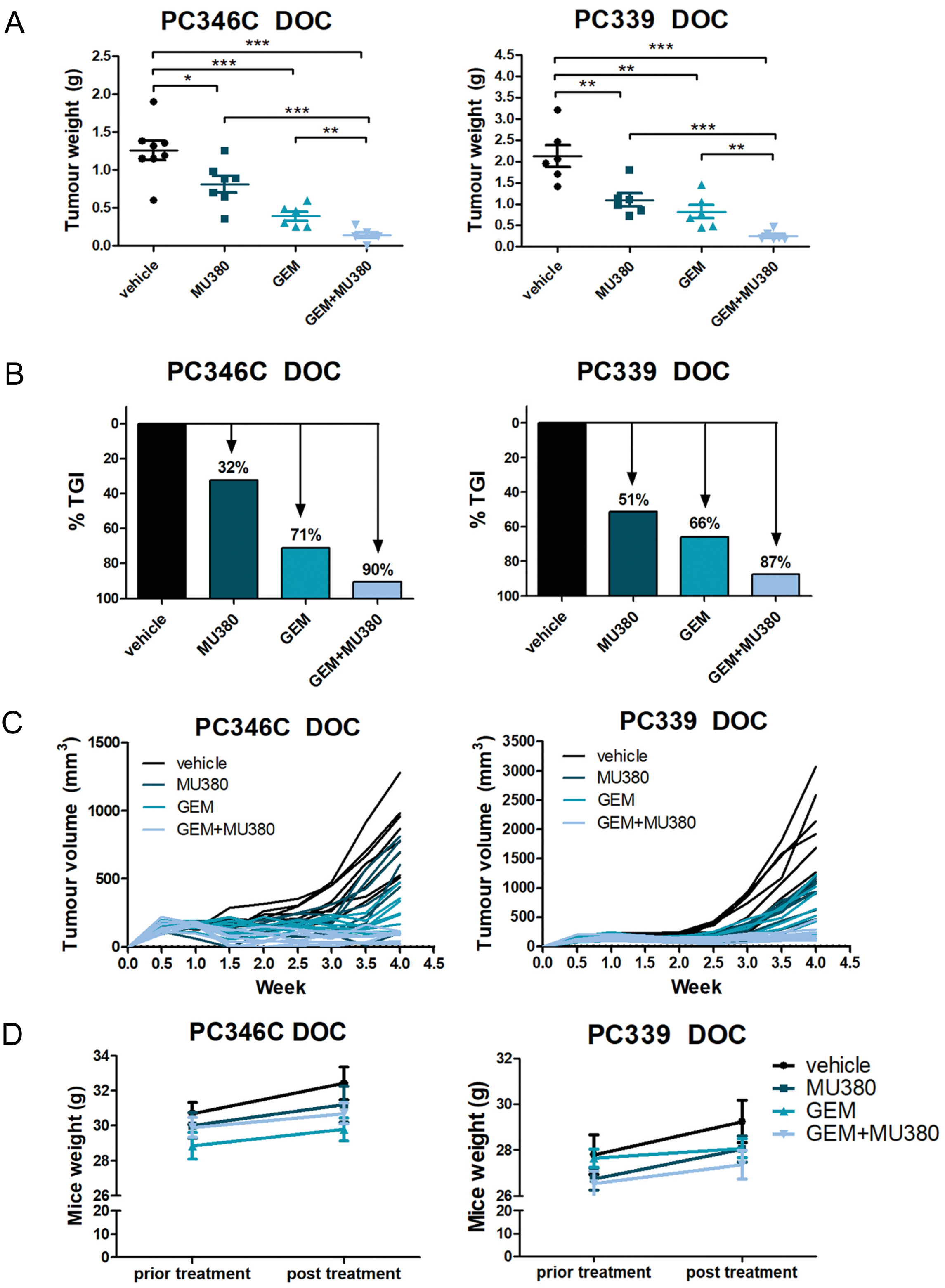


**Figure S10:** MU380 effectivity in docetaxel-resistant PC346C and PC339 in vivo xenograft models. A, Tumour weight analyzed at the endpoint of in vivo experiment. B, Tumor growth inhibition in response to various treatments. C, Single-tumor size curves displaying a response to various treatments for each mouse separately. Mice were treated with gemcitabine or MU380 alone or in combination on days 7, 14 and 21. D, Effect of the therapy on mice weight. Data represent means ± SEM (n≥6). ***, P<0.0001; **, P<0.01; *, P<0.05 by unpaired t-test. DOC, docetaxel-resistant; GEM, gemcitabine.
