# Supplementary material for: The CHK1 inhibitor MU380 significantly increases the sensitivity of human docetaxel‐resistant prostate cancer cells to gemcitabine through the induction of mitotic catastrophe
Source: Mol Oncol. 2020 Jul 16;14(10):2487–503. doi: 10.1002/1878-0261.12756 (PMC7530791; doi:10.1002/1878-0261.12756)
Supplement: Supplementary file 11 — Fig. S11. CHK1 inhibition potentiates the cytotoxic effect of gemcitabine. [file MOL2-14-2487-s011.pdf]

Figure S11

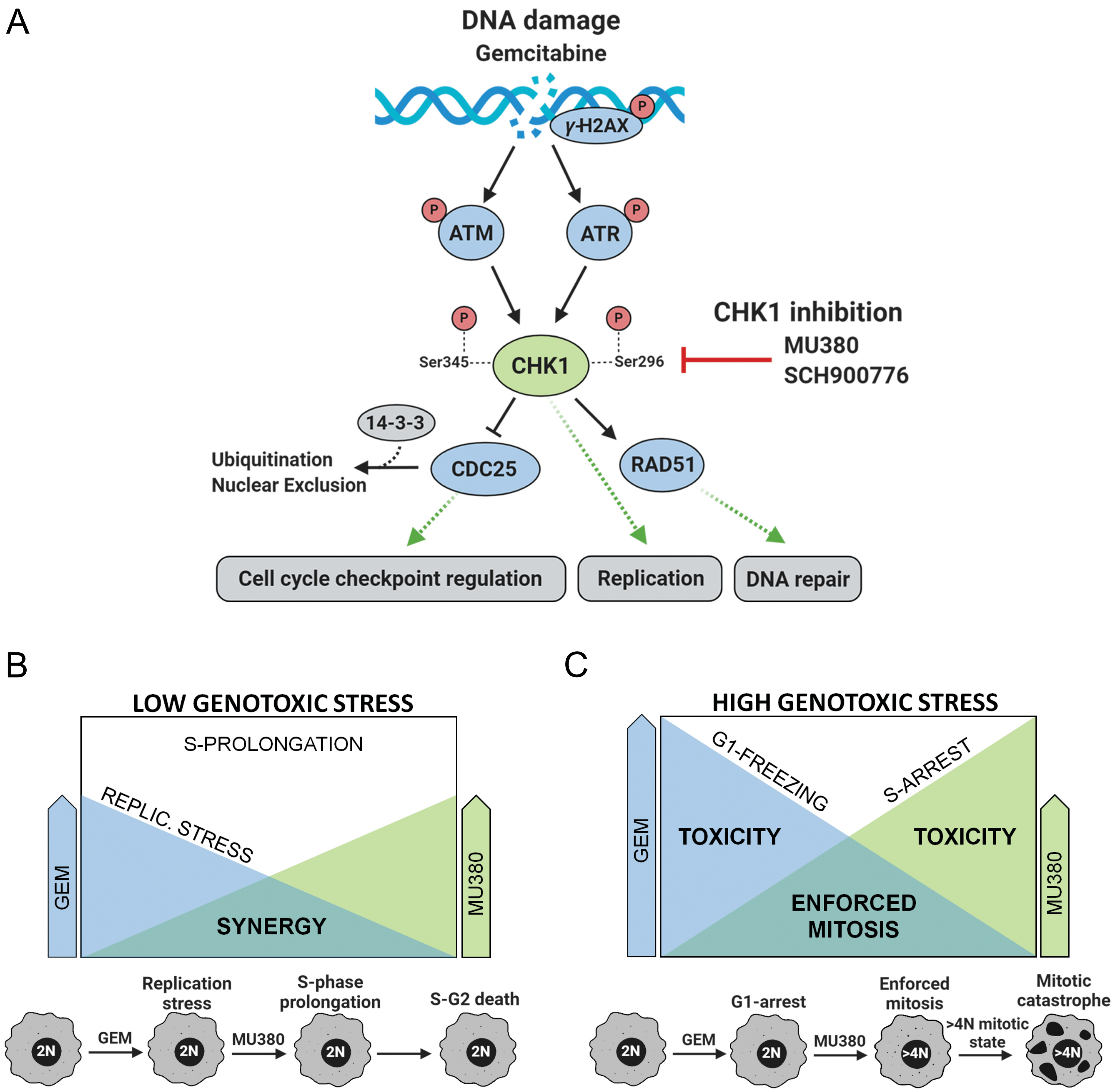

Figure S11: CHK1 inhibition potentiates the cytotoxic effect of gemcitabine. A, Illustration of the therapy approach exploiting Ser296 specific inhibition of CHK1 using MU380 or SCH900776 following gemcitabine-induced activation of DNA damage response pathway, which results in deregulation of cell cycle checkpoint, replication and DNA repair machinery (Isono et al., 2017; Italiano et al., 2018). B and C, Representative schemes illustrating the effect of gemcitabine in low (synergistic) (B) or high (C) concentration alone or in combination with MU380. Low gemcitabine dose leads to replicative stress and S-phase pro longation, persisting upon MU380 treatment and resulting in S-G2 cell death (Cappella et al., 2001; Koh et al., 2015). Conversely, genotoxic stress induced by the high dose of gemcitabine leads to massive G1-arrest (Pauwels et al., 2003), followed by enforced mitosis upon MU380 treatment eventually resulting in mitotic catastrophe (Koh et al., 2015). Created with Biorender.com.
