# Supplementary material for: The CHK1 inhibitor MU380 significantly increases the sensitivity of human docetaxel‐resistant prostate cancer cells to gemcitabine through the induction of mitotic catastrophe
Source: Mol Oncol. 2020 Jul 16;14(10):2487–503. doi: 10.1002/1878-0261.12756 (PMC7530791; doi:10.1002/1878-0261.12756)
Supplement: Supplementary file 12 — Table S1. Characteristics of PCa models. [file MOL2-14-2487-s012.docx]

| Supplementary Table 1: Characteristics of prostate cancer models. | | | | | | | | |  |
| --- | --- | --- | --- | --- | --- | --- | --- | --- | --- |
| Line |  |  | **Origin** | **Derived** | **AR** | **PSA** | **TP53** | **PTEN** | |
| DU145 | 1  2 | AG; DR  AG; DR | prostate | brain | - | - | +/- | +/- | |
| PC3 | 1  2 | AG; DR  AG; DR | prostate | bone | - | - | null | null | |
| PC346C |  |  | prostate | TURP | + | + | wt | wt | |
| PC346C-DOC |  |  | prostate | TURP | - | - | wt | wt | |
| PC339 |  |  | prostate | TURP | - | - | wt | mt | |
| PC339-DOC |  |  | prostate | TURP | - | - | wt | mt | |
| AR, androgen receptor; PSA, prostate-specific antigen; 1, models from Innsbruck; 2, models from Dublin; AG, aged control cells; DR and DOC, docetaxel resistant cells; TURP, transurethral resection of prostate; wt, wild type. | | | | | | | | |  |
