# Supplementary material for: The CHK1 inhibitor MU380 significantly increases the sensitivity of human docetaxel‐resistant prostate cancer cells to gemcitabine through the induction of mitotic catastrophe
Source: Mol Oncol. 2020 Jul 16;14(10):2487–503. doi: 10.1002/1878-0261.12756 (PMC7530791; doi:10.1002/1878-0261.12756)
Supplement: Supplementary file 13 — Table S2. IC50 values corresponding to dose‐response analysis from Fig. 1 and Fig. S4. [file MOL2-14-2487-s013.docx]

| Supplementary Table 2: IC_50_ values corresponding to dose-response analysis from Figure 1 and S3. | | | | | | |
| --- | --- | --- | --- | --- | --- | --- |
| Line | |  | **GEM** | **GEM+SCH900776** | **GEM+MU380** | **P-value** |
| DU145 AG 1 | IC_50_ (nM) | 26.9**^a^** | | 9.5**^b^** | 6.3**^c^** | <0.0001 |
|  | 95% CI of IC_50_ | 24.2 – 29.9 | | 8.1 – 11.2 | 5.2 – 7.5 |  |
| DU145 DR 1 | IC_50_ (nM) | 8.7**^a^** | | 5.1**^b^** | 1.9**^c^** | <0.0001 |
|  | 95% CI of IC_50_ | 8.0 – 9.3 | | 4.5 – 5.8 | 1.0 – 3.6 |  |
| PC3 AG 1 | IC_50_ (nM) | 23.7**^a^** | | 11.5**^b^** | 8.7**^b^** | <0.0001 |
|  | 95% CI of IC_50_ | 19.8 – 28.4 | | 9.4 – 13.9 | 7.4 – 10.1 |  |
| PC3 DR 1 | IC_50_ (nM) | 27.6**^a^** | | 24.4**^a^** | 9.6**^b^** | 0.0002 |
|  | 95% CI of IC_50_ | 23.3 – 32.6 | | 20.5 – 29.1 | 7.9 – 11.7 |  |
| DU145 AG 2 | IC_50_ (nM) | 28.5**^a^** | | 18.2**^b^** | 10.0**^c^** | <0.0001 |
|  | 95% CI of IC_50_ | 26.5 – 30.6 | | 16.9 – 19.5 | 9.3 – 10.9 |  |
| DU145 DR 2 | IC_50_ (nM) | 14.5**^a^** | | 10.5**^b^** | 5.5**^c^** | <0.0001 |
|  | 95% CI of IC_50_ | 12.2 – 17.1 | | 9.2 – 12.0 | 4.9 – 6.3 |  |
| PC3 AG 2 | IC_50_ (nM) | 10.2**^a^** | | 6.7**^b^** | 4.3**^c^** | <0.0001 |
|  | 95% CI of IC_50_ | 9.6 – 10.9 | | 6.2 – 7.1 | 4.0 – 4.6 |  |
| PC3 DR 2 | IC_50_ (nM) | 3.7**^a^** | | 3.3**^ab^** | 2.6**^b^** | 0.0398 |
|  | 95% CI of IC_50_ | 3.1 – 4.4 | | 2.7 – 4.0 | 2.2 – 3.1 |  |
| ^abc^ different letters denote statistically significant difference in IC_50_ between the treatment groups | | | | | | |
