# Supplementary material for: The CHK1 inhibitor MU380 significantly increases the sensitivity of human docetaxel‐resistant prostate cancer cells to gemcitabine through the induction of mitotic catastrophe
Source: Mol Oncol. 2020 Jul 16;14(10):2487–503. doi: 10.1002/1878-0261.12756 (PMC7530791; doi:10.1002/1878-0261.12756)
Supplement: Supplementary file 14 — Table S3. Overview of antibodies and other reagents used for immunoblotting, flow cytometry, imaging flow cytometry and immunostaining. [file MOL2-14-2487-s014.docx]

**Supplementary Table 3.** Overview of antibodies and other reagents used for immunoblotting, flow cytometry, imaging flow cytometry and immunostaining.

**Antibodies, materials and reagents used for western blot**

| **Antigen** | **Host** | **Dilution** | **Cat.No. (Producer)** |
| --- | --- | --- | --- |
| **CHK1** | Mouse | 1:1000 | sc-8408 (Santa Cruz) |
| **pCHK1 (Ser296)** | Rabbit | 1:500 | 2349 (Cell Signaling) |
| **pCHK1 (Ser345)** | Rabbit | 1:500 | 2348 (Cell Signaling) |
| **pH2AX (Ser139)** | Rabbit | 1:500 | 9718 (Cell Signaling) |
| **β-actin** | Mouse | 1:2000 | A5441 (Sigma) |
| **anti-Rabbit IgG1 HRP** | Donkey | 1:3000 | NA934V (Amersham) |
| **anti-Mouse IgG1 HRP** | Sheep | 1:4000 | NA931 (Amersham) |

| **Material** | **Cat.No.** | **Producer** |
| --- | --- | --- |
| **PVDF WB Membranes** | IPVH00010 | Millipore |
| **DC protein assay** | 500-0116 | Bio-Rad |
| **Immobilon Western Chemiluminescent HRP Substrate** | WBKLS0050 | Millipore |

| **RIPA buffer (homemade)**   - 790 mg and 900mg NaCl diluted in MQ (75mL), adjusted pH 7,4 - 10 mL of 10% Igepal in MQ water - 2,5mL of 10% Na-deoxycholate in MQ water - MQ water up to 100mL |
| --- |

| **Antigen** | **Conjugate** | **Host** | **Isotype and clonality** | **Dilution** | **Cat.No. (Producer)** |
| --- | --- | --- | --- | --- | --- |
| **p-H2A.X (Ser139)** | **PE** | Rabbit | IgG, clone DA1E | 1:50 | 5763 (Cell Signaling) |
|  | > ISO | Rabbit | IgG, clone DA1E | 1:50 | 5742S (Cell Signaling) |
| **RAD51** | **AF488** | Rabbit | IgG, EPR4030(3) | 1:300 | ab196449 (Abcam) |
|  | > ISO | Rabbit | IgG, EPR25A | 1:300 | ab199091 (Abcam) |
| **p-Histone H3 (Ser10)** | **biotin** | Rabbit | IgG, clone D2C8 | 1:50 | 3642 (Cell Signaling) |

**Antibodies and reagents used for multicolor analyses**

**Primary conjugated antibodies**

**Primary unconjugated antibodies**

| **Antigen** | **Conjugate** | **Host** | **Isotype and clonality** | **Dilution** | **Cat.No. (Producer)** |
| --- | --- | --- | --- | --- | --- |
| **α-tubulin** | unconjugated | Mouse | IgG1, DM1A | 1:50 | T9026 (Sigma) |
| **M30 CytoDEATH** | unconjugated | Mouse | IgG2b, M30 | 1:50 | 12140349001 (Roche) |

**Reagents used for secondary detection of unconjugated antibodies**

| **Streptavidin** | PerCP-eFluor710 |  |  | 1:1000 | 46-4317 (eBioscience) |
| --- | --- | --- | --- | --- | --- |
| **Streptavidin** | PE-Cy7 |  |  | 1:1000 | 405206 (Biolegend) |
| **Streptavidin** | PE |  |  | 1:1000 | 12-4317 (eBioscience) |
| **anti-mouse** | AF647 | Donkey | polyclonal | 1:1000 | A31571 (Invitrogen) |

| **Viability dyes** |  |
| --- | --- |
| LIVE/DEAD Green | L23101 (Molecular Probes) |
| LIVE/DEAD Violet | L34955 (Molecular Probes) |

| **Cell cycle dyes** |  |
| --- | --- |
| FxCycle Violet | F10347 (Thermofisher) |
| DAPI | A4099 (AppliChem) |

| **Other fluorescent probes** |  |
| --- | --- |
| Annexin V - FITC | ED7044, Exbio |
| Calcein AM | 89204, AnaSpec |
| Propidium iodide | P-4170, Sigma |
| TMRE | 1670392, Molecular Probes |
